# Intracellular self-assembly and metabolite analysis of key enzymes for L-lysine synthesis based on key components of cellulosomes

**DOI:** 10.3389/fmicb.2025.1596240

**Published:** 2025-06-16

**Authors:** Nan Li, Bowen Du, Xiankun Ren, Lu Yang, Peng Du, Piwu Li, Jianbin Wang, Junlin Li, Jing Xiao, Junqing Wang, Ruiming Wang

**Affiliations:** ^1^State Key Laboratory of Green Papermaking and Resource Recycling, Qilu University of Technology, Jinan, China; ^2^Dongxiao Biotechnology Co., Ltd., Zhucheng, China

**Keywords:** cellulosome, intracellular assembly, L-lysine, key enzyme gene, metabolite analysis

## Abstract

**Introduction:**

Cellulosome is a natural multi-enzyme complex in the extracellular space of anaerobic microorganisms, which has the advantages of small molecular weight, multiple binding sites, and strong designability. This study aimed to explore the influence of intracellular self-assembly complexes on L-lysine biosynthesis.

**Methods:**

Two novel L-lysine-engineered bacteria modification strategies were designed, considering the L-lysine biosynthesis pathway using DocA-S3/Coh as an efficient intracellular assembly element: pairwise assembly of key enzymes in cells and multi-enzyme assembly based on scaffolding proteins. Seven strains of key enzyme pairwise-assembled engineered bacteria were constructed, and four strains of multi-enzyme-assembled engineered bacteria were designed based on the scaffold protein genome.

**Results:**

The production of L-lysine by multi-enzyme-assembled engineered strain *Escherichia coli* QDE-*aspC*-DocA-S3-*lysC*:pET-28a-ScaA was 46.9% higher than that of *E. coli* QDE, and the conversion rate was increased from 50.9 to 59.8%. By combining specific analyses with metabolomics, 40 core metabolites of the assembled engineered bacteria were identified and mapped to L-lysine-related metabolic pathways, and the mechanism of how intracellular multi-enzyme assembly promoted the efficient synthesis of multiple amino acids was analyzed.

**Conclusion:**

This strategy exerts the “proximity effect” among multi-enzyme complexes, improves the transfer efficiency of intermediate metabolites between different catalytic active centers, indirectly improves the catalytic rate of each key enzyme, and provides a novel idea and technical platform for other multi-enzyme intracellular assemblies.

## 1 Introduction

Cellulose, hemicellulose, and lignin are components of plant biomass, and cellulose is a significant component of lignocellulose and a polysaccharide component of plant cell wall constituents, which are distributed in the biosphere (Gilbert, 2007; Pei et al., 2012; Shi et al., 2013; Shi et al., 2014). Cellulose-degrading bacteria, collectively referred to as bacteria, fungi, and actinomycetes, readily degrade natural cellulose (Adams et al., 2010). In recent years, anaerobic cellulose-degrading bacteria have been shown to produce cellulases, which assemble into cellulosomes, a condition facilitated by a specific functional skeleton. Cellulosomes are garnering attention as molecular machines for cellulose degradation (Xu et al., 2013; Nash et al., 2016; Bule et al., 2018). Generally produced by anaerobic microorganisms, cellulosomes are macromolecular complexes assembled by scaffold proteins and various enzymes acting as adhesion proteins that attach to the surface of anaerobic organisms and efficiently degrade hemicelluloses and cellulose (Shabat et al., 2016). The cellulosome complex has a size of 2 × 10^6^–6 × 10^6^ Da and comprises 14–50 subunits (Lamed et al., 1983). It is primarily divided into two parts: the catalytic subunit enzyme protein containing dockerin (Doc), which has catalytic function, and scaffoldins, a non-catalytic subunit containing the adhesive domain cohesion (Coh) of multiple types of adhesive modules with assembly function (Doi et al., 2003; Bayer et al., 2008; Tsai et al., 2022). The cellulosome of the gram-positive bacterium *Clostridium thermocellum* comprises two scaffold proteins and enzymes. The scaffold proteins consist of conventional type-I primary and type-II anchored scaffold proteins (Kosugi et al., 2004; Artzi et al., 2017). The scaffold protein contains nine Coh; the Coh of the primary scaffold protein is type I, while that of the anchor scaffold protein is type II (Sajjad et al., 2010; Noach et al., 2010). The connection formed with carbohydrate-binding module (CBM) is two Coh, followed by CBM3 and seven Coh (Xu et al., 2004).

Based on the framework of cellulosomes, You and Zhang (2013) of the Virginia Institute of Technology, United States, connected the continuous enzymes triosephosphate isomerase, aldolase, and fructose 1,6-bisphosphatase in the glycolysis pathway in tandem and constructed an anabolic pathway to verify the feasibility of assembling artificial cellulosomes, which has a good substrate channel effect. Jindou et al. (2014), Meijo University, Japan, constructed a synthetic pathway from SAM to ethylene in cyanobacteria using the cellulosome skeleton and increased the ethylene accumulation by 3.7 times through the substrate channel effect. Liu et al. (2013) from the University of Delaware in the United States used the artificial cellulosome framework to display three cascaded enzymes on the surface of yeast to oxidize methanol to carbon dioxide to produce NADH, and the reaction rate was increased five-fold. Lin et al. (2017) of the University of California, Riverside, the United States, used the artificial cellulosome framework to locate multiple enzymes on the surface of yeast lipid droplets, and the rate of producing ethyl acetate was twice as high as that of the enzymes without the cellulosome framework. Yang et al. (2018) used the artificial cellulosome framework to display lipase, carboxylate reductase, and acetaldehyde decarboxylase on the surface of *Yarrowia lipolytica* and the conversion rate of triglycerides to aliphatic hydrocarbons was increased by 17 times, while those of free enzymes were increased from 7 to 32% to 71 to 84%. Li et al. (2019) further immobilized lipase and P450 fatty acid decarboxylase on cellulose using the artificial cellulosome framework, which also realized the efficient conversion of triglycerides to aliphatic hydrocarbons, and the immobilized catalyst could be recovered and reused many times. Therefore, artificial cellulosomes can be applied to various microbial cells extracellularly to build different metabolic pathways to achieve a substantial increase in reaction rate or yield, with excellent practical application effects. Through the spatial proximity effect of enzymes, substrate channels are formed between cascade enzymes, which reduce the toxicity of intermediate products and the low effective substrate concentration of downstream enzymes caused by the diffusion of intermediate products.

L-lysine is produced via microbial fermentation. The primary bacterial strains are *Corynebacterium glutamicum* and *Escherichia coli*. Among the known microorganisms and plants with L-lysine biosynthesis pathways, the pathway comprises two distinct pathways: the aminoadipic acid pathway (AAA) and diaminoheptadecic acid pathway (DAP) (Eggeling and Sahm, 1999; Wittmann and Becker, 2007). In contrast to the AAA pathway, which primarily exists in higher fungi (such as yeast and molds) and archaea, the DAP pathway is primarily found in organisms such as bacteria and higher plants. L-lysine is formed in at least seven catalytic reaction steps using L-aspartic acid as the substrate. In *E. coli*, aspartic kinase (encoded by *lysC*), aspartic hemialdehyde dehydrogenase (encoded by *asd*), dihydropyridine dicarboxylate synthase (encoded by *dapA*), dihydropyridine dicarboxylate reductase (encoded by *dapB*), tetrahydropyridine succinylase (encoded by *dapD*), n-succinyl-L diaminopimelic acid aminotransferase (encoded by *dapC*), n-succinyl-L diaminopimelic acid desuccinylase (encoded by *dapE*), diaminopimelic acid differential isomerase (encoded by gene *dapF*), and diaminopimelic acid decarboxylation (encoded by *lysA*) (Velasco et al., 2002; Hermann, 2003) constitutes the *E. coli* DAP pathway (Dong et al., 2016; Xu et al., 2016). Using glucose as a substrate, L-lysine is produced through microbial fermentation. To increase L-lysine yield, improving the microorganisms via various methods is essential. The theoretical limitations of metabolic pathway modifications have been approached through genetic engineering. Therefore, improving the fermentation intensity of L-lysine and shortening the fermentation time based on the current conversion rates of sugar and acid have recently become a focus in industrial L-lysine production strain breeding (Sprenger, 2007; Stolz et al., 2007).

To construct self-assembled multi-enzyme complexes in cells, we used a semi-rational design and other methods in the early stage, combined with the self-constructed bimolecular fluorescence complementation-flow cytometry high-throughput screening system based on flow cytometry, to identify the effective cellulosome docking protein mutant DocA-S3 (Li et al., 2024). The assembling activity of the crucial components of cellulosomes in a microcalcium environment was determined, and the interaction between DocA and Coh was successfully realized in an intracellular environment. Based on this, L-lysine biosynthesis was further studied.

This study focused on addressing the challenge of “exploring the intracellular self-assembly mode of key enzymes for L-lysine synthesis.” For the L-lysine-producing bacterium *E. coli* QDE, glucose is used as the substrate and pyruvate is accumulated through glycolysis. After pyruvate is converted to oxoacetate, the role of aspartate aminotransferase (encoded by *aspC*) is strengthened, so that the anabolic flow to L-aspartate is maximized. The intracellular self-assembled complex was applied for the efficient assembling of the key enzymes involved in L-lysine synthesis, and changes in the fermentation products in different assembly modes and their potential mechanisms were investigated. The multi-enzyme complex proximity effect is utilized to enhance the synthesis efficiency. In contrast to traditional methods that primarily focus on promoters, Shine-Dalgarno sequence optimization, and positive transformation of key enzyme molecules in the L-lysine synthesis pathway, this method provides a new approach to improving the fermentation intensity of intracellular products (Sagong and Kim, 2017; Shang et al., 2017). To date, no relevant studies have been reported. This study has expanded the application field and range of intracellular multi-enzyme complexes based on the cellulosome model and provides a technology platform for the intracellular assembling of other multi-enzyme complexes.

## 2 Materials and methods

### 2.1 Strains and culture

#### 2.1.1 Strains, vectors, chemicals, media, and culture conditions

*E. coli* BL21 (DE3) was used as the expression host and cultured in a Luria Broth (LB) medium at 37°C. The gene was cloned into the pET28a(+) vector (Sangon, Shanghai, China). The enzymes used for DNA amplification and digestion were purchased from Vazyme (Nanjing, China). The primers were synthesized by Qingke (Beijing, China). Applications for the synthesis of multi-enzyme-assembled gene fragments were developed using GenScript. A multifragment seamless cloning and plasmid preparation kit was purchased from Vazyme. For metabolomics, we used Biotree (Shanghai, China). All chemicals were purchased from Sigma-Aldrich. The strains and plasmids used are listed in Table 1.

**Table 1:** Strains and plasmids used in this study.

Strain/plasmid	Relevant genotype	Source
Strain		
*E. coli* DH5α	Amplification and extraction of plasmids	Vazyme
*E. coli* BL21 (DE3)	Protein expression and extraction	Vazyme
*E. coli* QDE	L-lysine-producing bacteria, *∆ldcC*, *∆amiD*, *∆LYP1*, *icd*-D410E, *pykA*-G168D	This study
Plasmid		
pET-28a(+)-*aspC*/ *lysC*	Expression of protein AspC and LysC	This study
pET-28a(+)-*lysC*/ *asd*	Expression of protein LysC and Asd	This study
pET-28a(+)-*asd*/ *dapA*	Expression of protein Asd and DapA	This study
pET-28a(+)-*dapA*/ *dapB*	Expression of protein DapA and DapB	This study
pET-28a(+)-*dapB*/ *dapE*	Expression of protein DapB and DapE	This study
pET-28a(+)-*dapE*/ *dapF*	Expression of protein DapE and DapF	This study
pET-28a(+)-*dapF*/ *lysA*	Expression of protein DapF and LysA	This study
pET-28a(+)-*aspC*-Coh/ DocA-S3-*lysC*	Co-expressed DocA-S3-*aspC*/Coh-*lysC*	This study
pET-28a(+)-*LysC*-Coh/ DocA-S3-*asd*	Co-expressed DocA-S3-*lysC*/Coh-*asd*	This study
pET-28a(+)-*asd*-Coh/ DocA-S3-*dapA*	Co-expressed DocA-S3-*asd*/Coh-*dapA*	This study
pET-28a(+)-*dapA*-Coh/ DocA-S3-*dapB*	Co-expressed DocA-S3-*dapA*/Coh-*dapB*	This study
pET-28a(+)-*dapB*-Coh/ DocA-S3-*dapE*	Co-expressed DocA-S3-*dapB*/Coh-*dapE*	This study
pET-28a(+)-*dapE*-Coh/ DocA-S3-*dapF*	Co-expressed DocA-S3-*dapE*/Coh-*dapF*	This study
pET-28a(+)-*dapF*-Coh/ DocA-S3-*lysA*	Co-expressed DocA-S3-*dapF*/Coh- *ysA*	This study
pET-28a(+)-ScaA	Expression of scaffolding protein白ScaA	This study
pKD46	Expression of recombinant enzyme	This study
pKD13	Amplified FRT-kan-FRT fragment	This study
pCP20	Expression of flipping recombinase FLP	This study

### 2.2 Design strategy of the key enzyme assembly for L-lysine synthesis

Based on the mutant DocA-S3, the key enzyme genes *aspC*, *lysC*, *asd*, *dapA*, *dapB*, *dapE*, *dapF*, and *lysA* in the L-lysine fermentation pathway were considered as study objects, and the connection direction between the key enzymes and the cellulosome element DocA-S3/Coh was determined based on the protein structure. A location distant from the center of the catalytic activity of the key enzyme was selected. Two assembly strategies were used to study the intracellular assembly of the key cellulosome enzymes for L-lysine synthesis. First, the co-expression system of DocA-S3-aspC and Coh-lysC was constructed using free enzyme assembling, and eight enzymes were assembled in pairs. The seven key enzyme gene combinations were *aspC* and *lysC*, *lysC* and *asd*, *asd* and *dapA*, *dapA* and *dapB*, *dapB* and *dapE*, *dapE* and *dapF*, and *dapF* and *lysA*. Similarly, based on the multi-enzyme assembling method of scaffold proteins, nine repeats of adhesion protein Coh constituted scaffold protein ScaA for expression, and the intracellular assembling of nine key enzymes for L-lysine synthesis was performed once. The four key combined enzyme genes were *aspC* and *lysC*, *asd* and *dapA*, *dapA* and *dapB*, and *dapE* and *dapF*. Through *E. coli* Red homologous recombination technology, we achieved efficient integrated editing of chromosome genes based on λphage Red recombinase. The docking protein mutant DocA-S3 and key L-lysine synthesis enzymes were sequentially assembled in the genome and transferred into the truncated scaffold protein ScaA (Coh) for expression.

### 2.3 Construction of pairwise-assembled strains of key enzymes and the fermentation method

#### 2.3.1 Construction of the assembly plasmid

**Figure 1:**
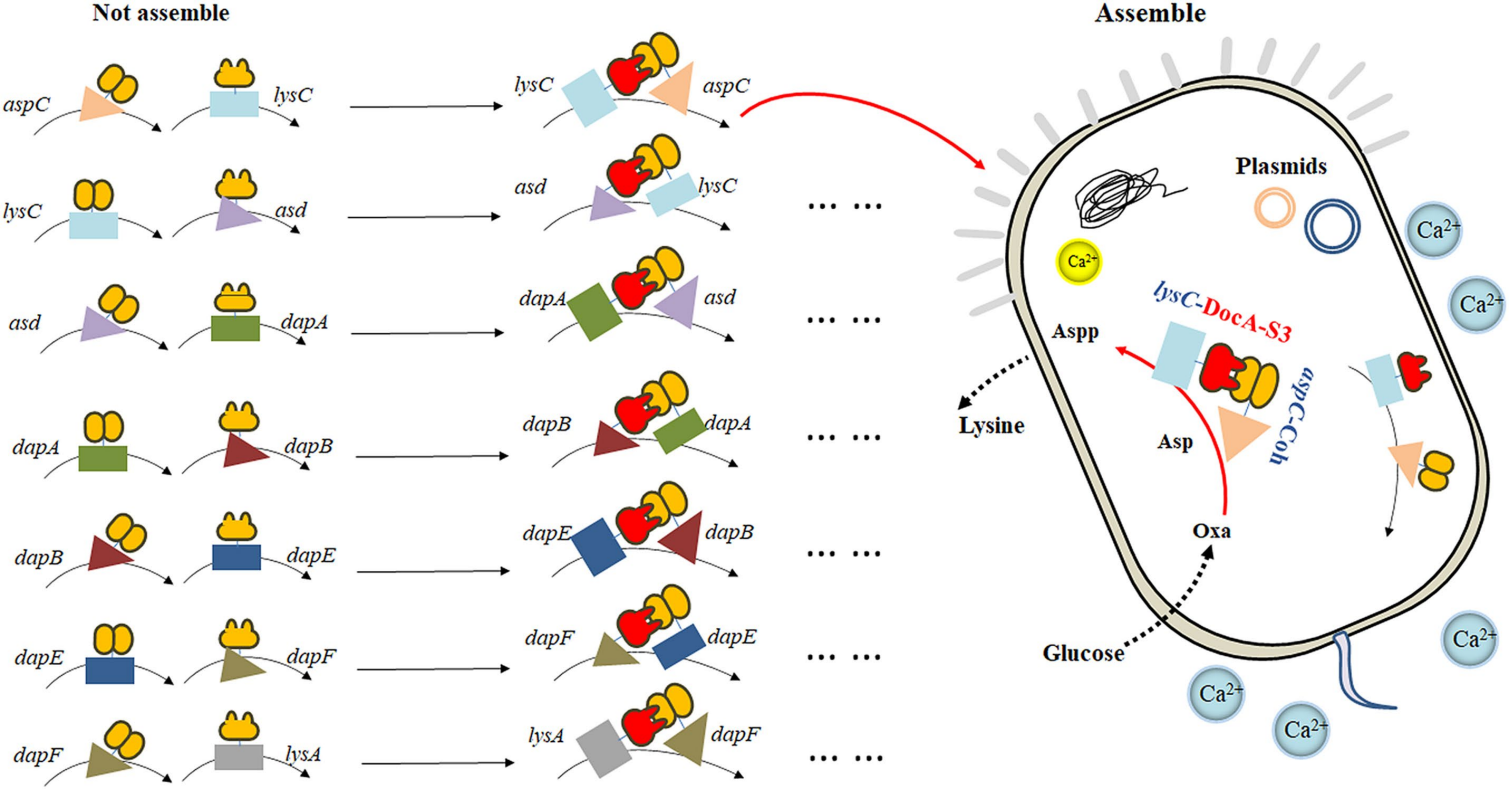
Schematic diagram of the pairwise assembly of L-lysine key enzymes.

The key cellulosome components, the DocA-S3 mutant and Coh, were linked to the key enzyme genes *aspC* and *lysC* in the L-lysine fermentation pathway by the linking peptide SGGGSGGGSGGGS, respectively: according to the promoter, pathway enzyme gene, flexible linking peptide, cellulosome Coh, and terminator. The promoter, pathway enzyme gene, flexible ligand peptide, cellulosome DocA-S3, and terminator were assembled and cloned into pET-28a(+) (Ptrc) for co-expression. The pairwise assembly of the eight enzymes was cloned into the plasmid expression vector. The design of the key enzymes for L-lysine synthesis in the intracellular pairwise assembly is shown in Figure 1. The recombinant plasmid was transferred into *E. coli* QDE-producing L-lysine, and seven engineered strains were obtained. Control strains lacking the cellulosome element Coh were obtained via reverse polymerase chain reaction (PCR), and a pairwise overexpression of key enzyme genes in the pathway was achieved only through plasmid optimization. The primers used are presented in Supplementary Table S1. Primers were synthesized by Shenggong Biology (Shanghai).

#### 2.3.2 Shaking flask fermentation culturing method of strain

The conventional strain activation method, plate streaking, was adopted, and culturing was conducted in a constant temperature incubator (SHHB-D6000 Serials; Zhengzhou Shengyuan Instrument Co., Ltd., Zhengzhou, China) at 37°C. The culture was shaken at 37°C, inoculated at 14–15%, 200 × *g*, maintained at the stable pH 7.0 of the culture solution, and fermented for 36–40 h. The changes in pH optical density at 600 nm (OD_600_) were monitored.

### 2.4 Construction and fermentation method of multi-enzyme assembly strain based on scaffold protein

#### 2.4.1 Design of primers for red homologous recombination

The scaffold protein ScaA comprising nine repeated adhesion proteins and Coh was expressed using the carrier plasmid pET-28a(+). The key combined enzyme genes *aspC* and *lysC*, *asd* and *dapA*, *dapA* and *dapB*, and *dapE* and *dapF* in the L-lysine fermentation pathway and the docking protein mutant DocA-S3 were selected for double-fragment homologous assembly in the genome. *E. coli* genomic DNA was extracted, and the pKD46, pKD13, and pCP20 plasmids were extracted.

According to the gene sequence of L-lysine-producing bacteria *E. coli* QDE genome sequencing, the genes encoding key enzymes (*aspC*, *LysC*, *asd*, *dapA*, *dapB*, *dapE*, and *dapF*) were searched, and the upstream and downstream homologous arm primers were designed using Oligo 7.0 software. The upstream and downstream primers *X*-S3-F/R of the DocA-S3 gene were linked to the assembly gene. The size of the FRT locus fragment on plasmid pKD13 was 1,324 bp, and the upstream and downstream primers FRT-*X*kan-FRT-F/R were the FRT-Xkan-FRT locus fragments (FRT-XkAN-FRT) overlapping with the homologous arm of the assembly gene. Upstream and downstream primer assembly frames X-F/R; upstream and downstream validation primers Ypkd46-F/R for the pKD46 plasmid; upstream and downstream validation primers Ypcp20-F/R for the pCP20 plasmid. The primer sequences are listed in Supplementary Table S2.

#### 2.4.2 Acquisition of homologous recombination assembly fragments

Considering the assembly of *aspC*, a key enzyme for L-lysine synthesis, the gene homologous arm was constructed using PCR to amplify the four gene fragments required for the construction of the intracellular assembly frame. First, *aspC*1-F and *aspC*1-R were used to clone the upstream homologous arm *aspC1* of the assembled *aspC*. *aspC*2-F and primer *aspC*2-R were used to clone and assemble the downstream homologous arm *aspC*2 of *aspC*. Using the upstream and downstream primers *aspC*-DocA-S3-F and *aspC*-DocA-S3-R, respectively, we cloned the DocA-S3 fragment connected to the *aspC* homologous arm. The upstream primer *aspC*-FRT-F and downstream primer *aspC*-FRT-R were used to clone the Kan-resistant FRT locus fragment (FRT-*aspC* Kan-FRT) on the pKD13 plasmid, which were linked to the homologous arm of DocA-S3 and *aspC*. The amplification system is presented in Table 2.

**Table 2:** Gene polymerase chain reaction association amplification system configuration.

Reagent	Volume (μL)
2 × phanta	25
Upstream primer	2
Downstream primer	2
ddH_2_O	19
Template	2
Total	50

#### 2.4.3 Construction of key enzyme gene-multi-fragment seamless cloning assembly frame

The upstream homologous arm *aspC*1, downstream homologous arm *aspC*2, docking protein mutant fragment *aspC*-DocA-S3, and FRT-*aspC* Kan-FRT site fragment of *aspC* were obtained by combining the procedures summarized in Sections 2.4.1 and 2.4.2. Multi-fragment seamless cloning was performed using the linearized vector pET-28a(+). Thus, the four genes were linked to obtain the *aspC* assembly frame *aspC*1-DocA-S3-FRT-*aspC* Kan-FRT-*aspC*2, and the FRT locus fragment containing the resistance was placed in the middle.

#### 2.4.4 Multi-enzyme assembly on the genome

The temperature-sensitive plasmid pKD46 required for Red recombination was transformed into *E. coli* QDE. After recovery, single colonies were selected and inoculated in the medium supplemented with 100 μL/mL ampicillin antibiotic and cultured at 30°C and 200 × *g*. The bet gene on the pKD46 plasmid was amplified using YpKD46-F and YpKD46-R as the upstream and downstream primers, respectively.

*E. coli* QDE-pKD46 was induced to express recombinant protein using 10 mmol/L L-arabinose and cultured to an OD_600_ of 0.2–0.6. The key enzyme gene assembly frame fragments obtained in Section 2.4.3 through electrotransfer of receptive cells were prepared and cultured with a 100 μL/mL ampicillin antibiotic and 50 μL/mL kanamycin medium and verified via culturing at 30°C and 200 × *g*. The culture temperature was increased to 42°C, and the pKD46 plasmid was eliminated in a non-resistant LB liquid medium cultured at 200 × *g* for 12 h. L-lysine-producing bacteria with gene assembly frame fragments of the key enzymes in genome integration were obtained.

The thermosensitive plasmid pCP20 was transformed to eliminate the Kan resistance fragment in the assembly frame of the key enzyme gene of the engineered bacteria, and the CmR gene on the pCP20 plasmid was amplified using YpCP20-F and YpCP20-R as upstream and downstream primers, respectively. The culture temperature was increased to 42°C, and the resistant LB liquid medium was cultured at 200 × *g* for 12 h to eliminate the pCP20 plasmid. QDE-*aspC*-DocA-S3 was obtained via resistance verification. The relevant validation primers are listed in Supplementary Table S3. The steps in Sections 2.4.1–2.4.4 were repeated to complete the assembly of *aspC*, *lysC,* and docking protein mutant DocA-S3 at the genomic level, and Red recombinant-engineered bacteria QDE-*aspC*-DocA-S3-*LysC* was obtained.

QDE-*asd*-DocA-S3-*dapA*, QDE-*dapA*-DocA-S3-*dapB,* and QDE-*dapE*-DocA-S3-*dapF* were constructed as previously described.

#### 2.4.5 Electrical transformation of scaffold protein recombinant plasmid pET-28a(+)-ScaA

**Figure 2:**
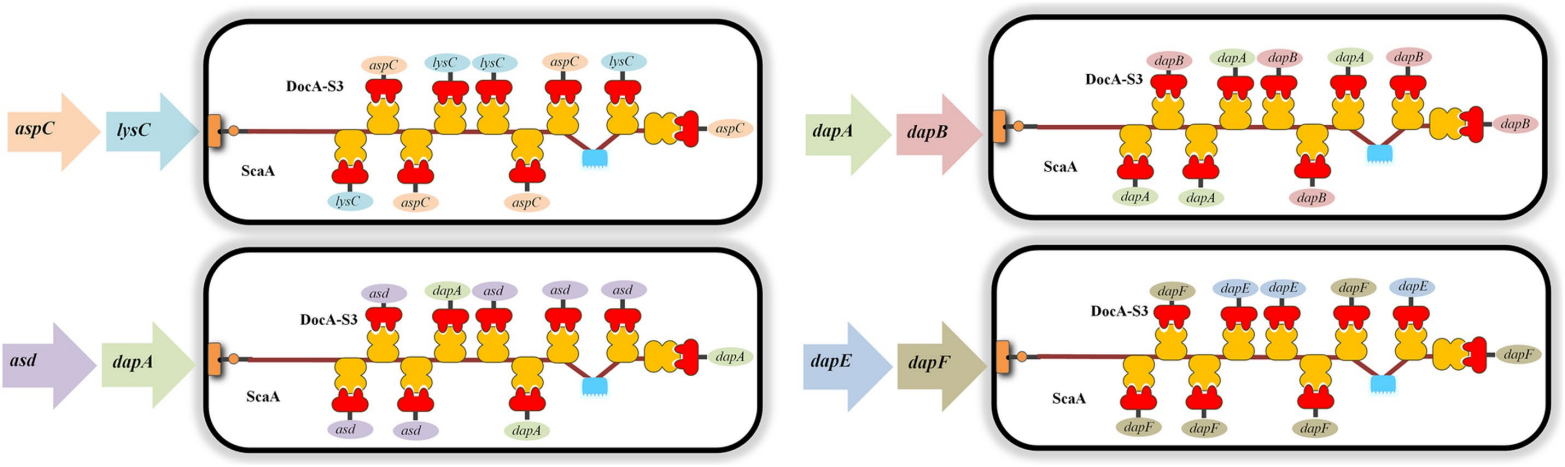
Schematic diagram of multi-enzyme assembly based on scaffold protein.

According to the basic skeleton of the intracellular assembly of cellulosomes, the plasmid pET-28a(+)-ScaA was electrically transformed into an engineered strain that was verified correctly as described in Section 2.4.4, and the multi-enzyme-assembled engineered bacteria based on the scaffold protein were finally obtained. Based on Red homologous recombination, the design idea for the multi-enzyme assembly of key enzymes for L-lysine synthesis based on scaffold proteins is shown in Figure 2.

#### 2.4.6 Fermentation of bacteria assembling key enzymes for L-lysine synthesis

Fermentation conditions: 37°C; pH 6.9; initial glucose concentration, 2.0%; dissolved oxygen, 20–36%. The L-lysine fermentation process adopted nutrient flow-adding technology, primarily involving the addition of glucose and ammonium sulfate solutions.

Control of dissolved oxygen in the seed liquid fermenter: dissolved oxygen control: 20–35%; when the dissolved oxygen was less than 25%, the rotational speed was successively increased to 1,000 × *g*; OD_600_ = 12–17 inoculation; culture cycle: 10 h.

The 5-L fermenter control scheme: 15% inoculation amount; pH: ammonia water pH was adjusted to 6.9 before inoculation; dissolved oxygen calibration: saturated sodium sulfite solution calibration 0 points, air calibration 100%; temperature: 37°C; pH: 6.9 ± 0.02. Initial conditions: temperature 37°C, pH 6.9, tank pressure 0.06 MPa, air volume 0.5 m^3^/h, speed 300 × *g*. Dissolved oxygen control: 25–37%; when the dissolved oxygen was less than 25%, the rotational speed was increased successively. Flow-adding control scheme: flow-adding sugar was 50–53% glucose; ammonium sulfate solution preparation: 80% ammonium sulfate. The fermentation experiments of different assembled engineering bacteria were set up in three parallel groups, and the fermentation time was 36 h. During the fermentation process, samples were collected every 4 h, and the absorbance of the diluted samples at 600 nm was measured using an ultraviolet spectrophotometer (WFJ 7200; Shanghai Uniko Instrument Co., Ltd., Shanghai, China) to determine the change in bacterial concentration. Glucose and L-lysine contents were determined using a biosensor (SBA-40E; Institute of Biology, Shandong Academy of Sciences, Jinan, China). A series of L-lysine standards of known concentrations were used. After preheating the machine, the standards were injected in sequence. The instrument automatically calibrated. The determination was repeated until the results were stable and the relative error was less than 1%, completing the calibration.

### 2.5 Analysis of the metabolites of L-lysine synthesis key enzyme of intracellular self-assembled-engineered bacteria

#### 2.5.1 Collection of experimental samples

There were seven strains of cellulosome skeleton-intracellular pairwise assembly key enzymes and four of cellulosome skeleton-intracellular multi-enzyme assembly key enzymes based on scaffold proteins. The culture medium was shaken, quickly removed, and centrifuged at 4°C (1,000 × *g*, 10 min). The supernatant (500 μL) was collected in a new centrifugal tube, and the liquid nitrogen was quenched for 30 s and stored at −80°C. To ensure data accuracy, the experimental samples were set in parallel thrice (Silas and Boas, 2005; Meyer et al., 2013; Pinu et al., 2017).

#### 2.5.2 Extraction of fermentation metabolites

The configuration of methanol: acetonitrile = 1:1 (v/v); an isotope internal standard was added as the extraction solution. The 100-μL sample was placed in a special centrifugal tube, and 400 μL of extraction solution was added. Subsequently, the sample was subjected to ultrasonic treatment in an ice water bath for 10 min after 30 s of vortex mixing and stored at −40°C for 1 h. The sample was centrifuged at 4°C and 1,2000 × *g* for 15 min for detection (Mashego et al., 2007; Wiklund et al., 2008; Dunn et al., 2011).

#### 2.5.3 Detection and data processing

Using the Vanquish (Thermo Fisher Scientific) ultra-high performance liquid chromatograph, Chromatographic separation of the target compounds was performed using Waters ACQUITY UPLC BEH Amide 2.1 mm × 100 mm, 1.7 μm liquid chromatography column (Wang et al., 2016). Based on ultra high-performance liquid chromatography-mass spectrometry using high-resolution mass spectrometry Orbitrap Exploris series instruments, the original data were converted into mzXML format using ProteoWizard software and processed with a self-written R program package (kernel XCMS) for peak identification, peak extraction, peak alignment, and integration (Smith et al., 2006). The metabolism of amino acids, sugars, and organic acids during L-lysine fermentation was analyzed to determine the complete changes in metabolite accumulation.

## 3 Results

### 3.1 Construction of cellulosome element- L-lysine synthetase intracellular pairwise assembly engineering bacteria

The eight enzymes were assembled in pairs and cloned into a plasmid expression vector. The seven key enzyme gene combinations were *aspC* and *lysC*, *lysC* and *asd*, *asd* and *dapA*, *dapA* and *dapB*, *dapB* and *dapE*, *dapE* and *dapF*, and *dapF* and *lysA*. The pairwise assembly diagram of the plasmids is shown in Supplementary Figure S1A (exemplified by the construction of recombinant plasmids of two key enzyme genes, *aspC* and *lysC*). Subsequently, the recombinant plasmid was transferred into the L-lysine-producing *E. coli* QDE. Seven engineered strains were obtained, including QDE:pET-28a(+)-*aspC*-Coh/DocA-S3-*lysC*, QDE:pET-28a(+)-*lysC*-Coh/DocA-S3-*asd*, QDE:pET-28a(+)-*asd*-Coh/DocA-S3-*dapA*, QDE:pET-28a(+)-*dapA*-Coh/DocA-S3-*dapB,* QDE:pET-28a(+)-*dapB*-Coh/DocA-S3-*dapE*, QDE:pET-28a(+)-*dapE*-Coh/DocA-S3-*dapF,* and QDE:pET-28a(+)-*dapF*-Coh/DocA-S3-*lysA*. Unassembled control engineered bacteria with pairwise overexpression of the key enzyme plasmids were obtained via reverse PCR. There were seven strains, namely QDE:pET-28a(+)-*aspC*/*lysC*, QDE:pET-28a(+)-*lysC*/*asd*, QDE:pET-28a(+)-*asd*/*dapA*, QDE:pET-28a(+)-*dapA*/*dapB*, QDE:pET-28a(+)-*dapB*/*dapE*, QDE:pET-28a(+)-*dapE*/*dapF,* and QDE:pET-28a(+)-*dapF*/*lysA*, that displayed only pair-to-pair overexpression of the key enzymes of L-lysine synthesis. The flowchart of the plasmid construction is shown in Supplementary Figure S1.

**Figure 3:**
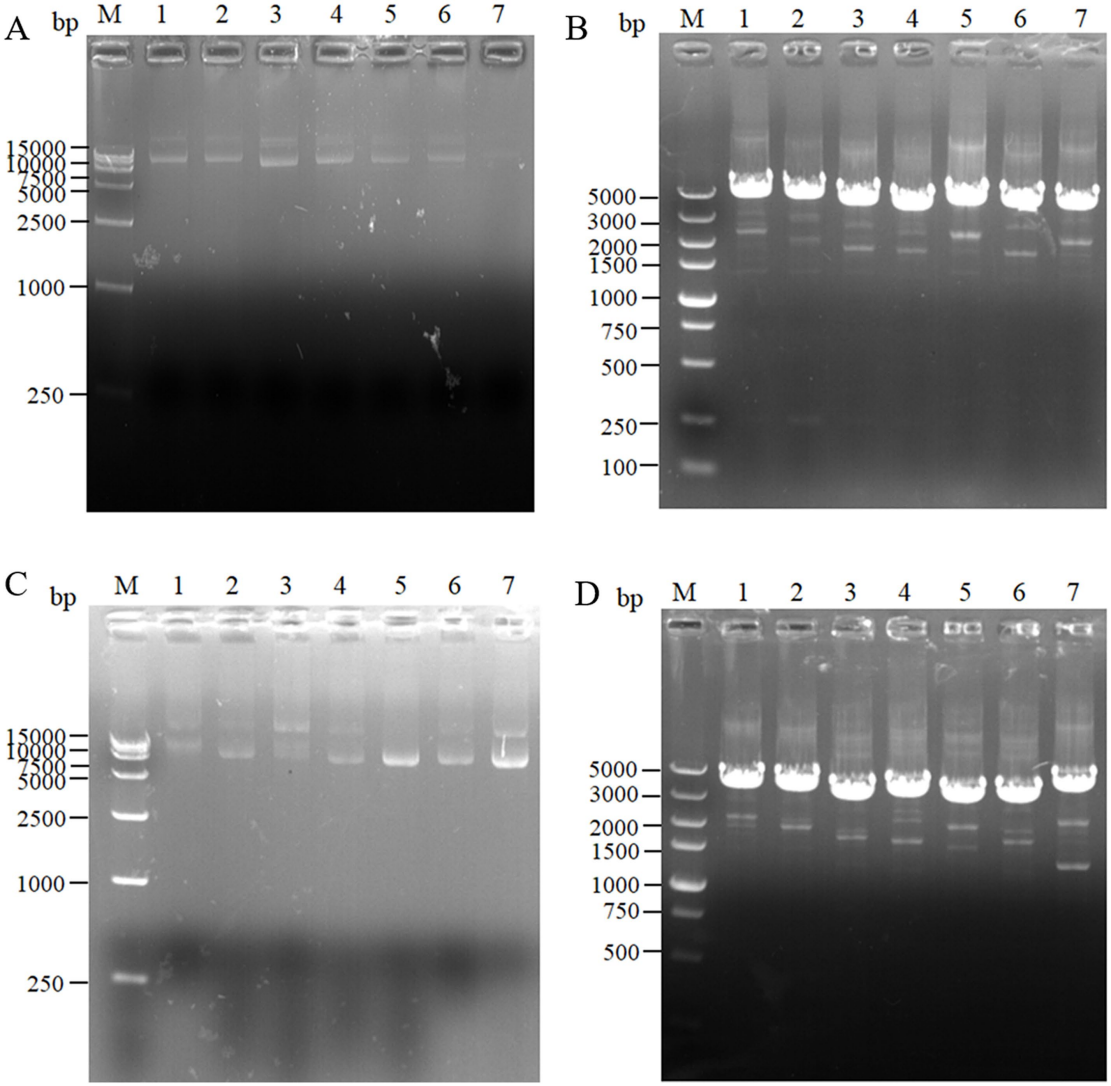
Agarose gel electrophoresis of pairwise assembly and validation. **(A)** 1: 9637 bp DNA of pET-28a(+)-*aspC*-Coh/DocA-S3-*lysC*; 2: 9550 bp DNA of pET-28a(+)-*lysC*-Coh/DocA-S3-*asd*; 3: 9079 bp DNA of pET-28a(+)-*asd*-Coh/DocA-S3-*dapA*; 4: 8797 bp DNA of pET-28a(+)-*dapA*-Coh/DocA-S3-*dapB*; 5: 9046 bp DNA of pET-28a(+)-*dapB*-Coh/DocA-S3-*dapE*; 6: 9049 bp DNA of pET-28a(+)-*dapE*-Coh/DocA-S3-*dapF*; 7: 9184 bp DNA of pET-28a(+)-*dapF*-Coh/DocA-S3-*lysA*; **(B)** Colony polymerase chain reaction (PCR) confirmed the transformation of assembled plasmids; **(C)** 1: 9148 bp DNA of pET-28a(+)-*aspC*/*lysC*; 2: 9061 bp DNA of pET-28a(+)-*lysC*/ *asd*; 3: 8590 bp DNA of pET-28a(+)-*asd*/*dapA*; 4: 8308 bp DNA of pET-28a(+)-*dapA*/*dapB*; 5: 8557 bp DNA of pET-28a(+)-*dapB*/*dapE*; 6: 8560 bp DNA of pET-28a(+)-*dapE*/*dapF*; 7: 8695 bp DNA of pET-28a(+)-*dapF*/*lysA*; **(D)** Colony PCR confirmed the transformation of unassembled control plasmids.

For the intracellular pair-to-pair assembly of eight key L-lysine enzymes, the synthetic plasmid was extracted to verify its size, and the electrophoretic results are shown in Figure 3A. The above plasmids were transformed into *E. coli* QDE, and the colony PCR verifications are shown in Figure 3B. The gene strip sizes in Lanes 1–7 were 4,629, 4,542, 4,071, 3,789, 4,984, 4,041, and 4,984 bp, respectively. Correspondingly, to obtain negative control plasmids without pairwise assembly within the cell, only pairwise overexpression of the key enzyme in L-lysine synthesis was performed. Reverse PCR priming was designed to remove the adhesion protein Coh required for assembly and obtain the remaining plasmids for cyclization. The electrophoresis results are shown in Figure 3C. The control plasmids were transformed into *E. coli* QDE and the colony PCR verifications are shown in Figure 3D. The gene strip sizes in Lanes 1–7 were 4,140, 4,053, 3,582, 3,300, 3,549, 3,552, and 3,687 bp. The strip was without impurities, and its length was consistent.

### 3.2 Construction of L-lysine synthesis multi-enzyme intracellular genome horizontal assembly engineered bacteria based on scaffold proteins

**Figure 4:**
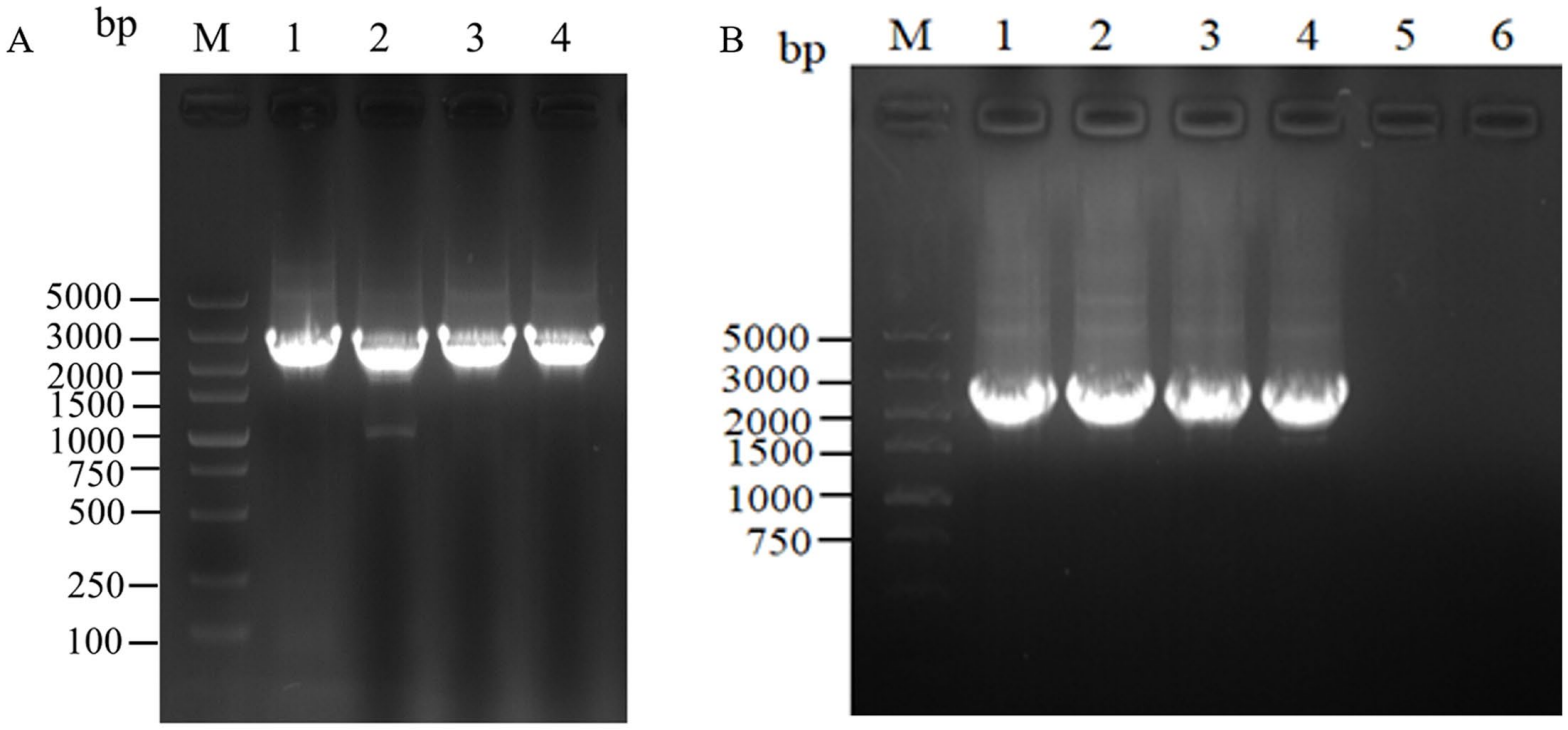
Electrophoretic images of the gene fragments of the assembly frames for the key enzyme of L-lysine synthesis. **(A)** 1: 2476 bp DNA of the assembly frame *aspC*1*-*DocA-S3-FRT-*aspC*Kan-FRT*-aspC*2 fragment; 2: 2476 bp DNA of the assembly frame *asd*1*-*DocA-S3-FRT-*asd*Kan-FRT*-asd*2 fragment; 3: 2476 bp DNA of the assembly frame *dapA*1*-*DocA-S3-FRT-*dapA*Kan-FRT*-dapA*2 fragment; 4: 2476 bp DNA of the assembly frame *dapE*1*-*DocA-S3-FRT-*dapE*Kan-FRT*-dapE*2 fragment; **(B)** 1: 2428 bp DNA of the assembly frame *lysC*1*-*DocA-S3-FRT-*lysC*Kan-FRT*-lysC*2 fragment; 2: 2428 bp DNA of the assembly frame *dapA*1*-*DocA-S3-FRT-*dapA*Kan-FRT*-dapA*2 fragment; 3: 2428 bp DNA of the assembly frame *dapB*1*-*DocA-S3-FRT-*dapB*Kan-FRT*-dapB*2 fragment; 4: 2428 bp DNA of the assembly frame *dapF*1*-*DocA-S3-FRT-*dapF*Kan-FRT*-dapF*2 fragment.

For the intracellular assembly of *aspC* and *lysC*, *asd* and *dapA*, *dapA* and *dapB*, and *dapE* and *dapF* based on scaffold proteins, double-fragment homologous assembly with the docking protein mutant DocA-S3 was performed sequentially. The assembling of *aspC*, *asd*, *dapA,* and *dapE* was performed sequentially according to the Red homologous recombination series. The assembly frame fragments of *aspC*, *asd*, *dapA*, and *dapE* were obtained through seamless cloning, and the electrophoresis results are shown in Figure 4A. The strip was without impurities, and its length was consistent. The assembly frame fragments were successively transferred into the receptor state of L-lysine-producing bacteria QDE-pKD46, the thermosensitive plasmid pKD46 was eliminated, and the plasmid pCP20 was transferred to identify FRT sites and eliminate Kan resistance in FRT-KAN-FRT in the genome. The electrophoretic verification is shown in Supplementary Figure S2. Four engineered strains, QDE-*aspC*-DocA-S3, QDE-*asd*-DocA-S3, QDE-*dapA*-DocA-S3, and QDE-*dapE*-DocA-S3 were obtained by eliminating the temperature-sensitive plasmid pCP20.

The assembling of *lysC*, *dapA*, *dapB,* and *dapF* was continued according to the Red homologous recombination series of steps, and the electrophoretic results are shown in Figure 4B. Four Red homologous recombinant-engineered strains, QDE-*aspC*-DocA-S3-*lysC*, QDE-*asd*-DocA-S3-*dapA*, QDE-*dapA*-DocA-S3-*dapB,* and QDE-*dapE*-DocA-S3-*dapF* were obtained. The electrophoretic verification is shown in Supplementary Figure S3.

**Figure 5:**
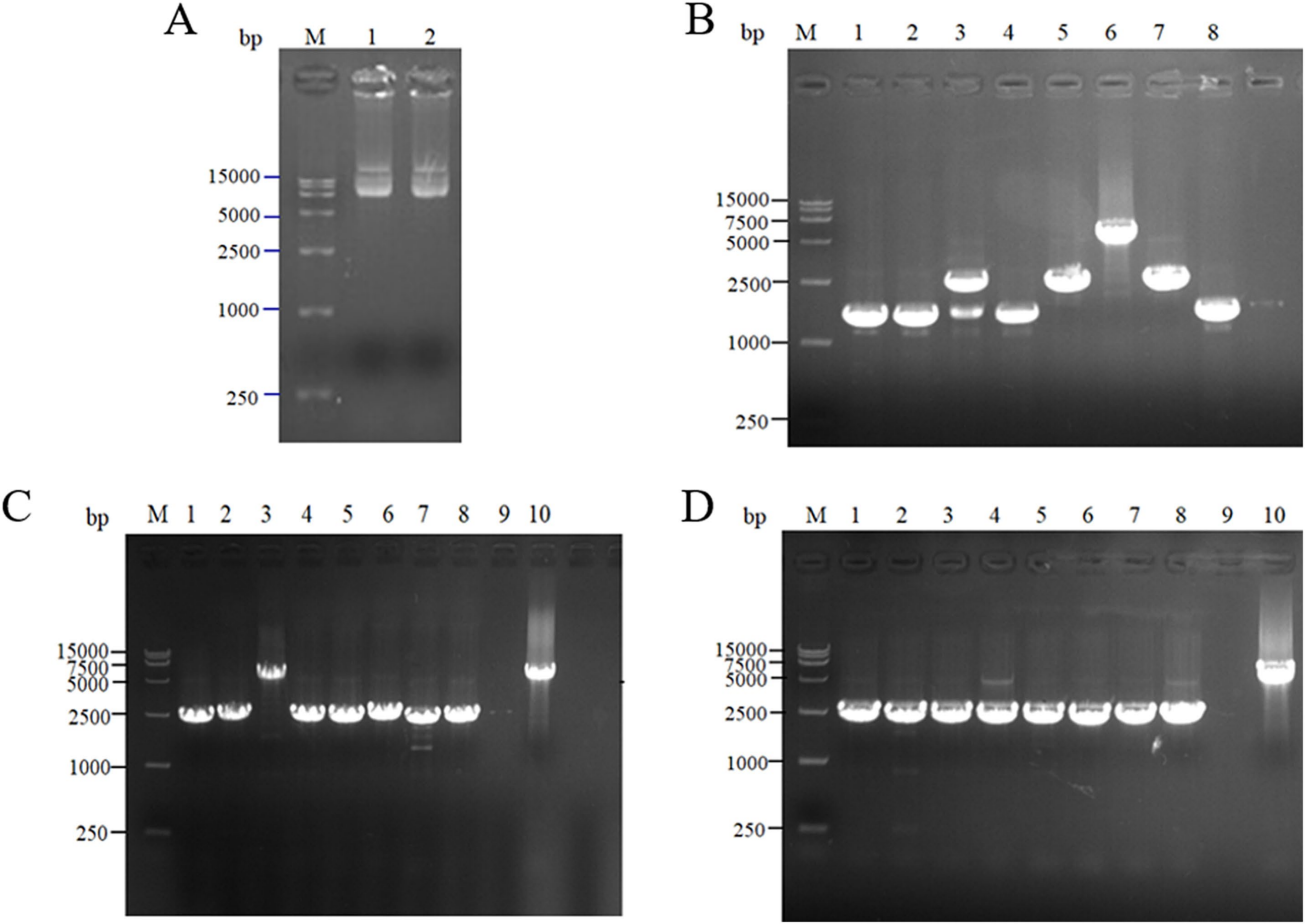
Expression of scaffold protein ScaA. **(A)** 1–2: 1,0132 bp DNA of pET-28(+)-ScaA; **(B)** 1–8: 5100 bp DNA of QDE-*aspC*-DocA-S3-*lysC*:pET-28a-ScaA fragment verification; **(C)** 1–5: 5100 bp DNA of QDE-*asd*-DocA-S3-*dapA*:pET-28a-ScaA fragment verification; 6–10: 5100 bp DNA of QDE-*dapA*-DocA-S3-*dapB*:pET-28a-ScaA fragment verification; **(D)** 1–10: 5100 bp DNA of QDE-*dapE*-DocA-S3-*dapF*:pET-28a-ScaA fragment verification.

The scaffold protein-based multi-enzyme assembly involved the cellulosome element; therefore, the scaffold protein ScaA was designed to be continuously expressed with nine Coh proteins and cloned into pET-28a(+) for expression. The electrophoresis results for the pET-28(+)-ScaA plasmid are shown in Figure 5A. The plasmid was transformed into the engineered bacteria QDE-*aspC*-DocA-S3-*lysC*, QDE-*asd*-DocA-S3-*dapA*, QDE-*dapA*-DocA-S3-*dapB,* and QDE-*dapE*-DocA-S3-*dapF*, and the results are shown in Figures 5B–D. The strip was without impurities, and its length was consistent. Four strains of multi-enzyme assembled engineered bacteria QDE-*aspC*-DocA-S3-*lysC*:pET-28a-ScaA, QDE-*asd*-DocA-S3-*dapA*:pET-28a-ScaA, QDE-*dapA*-DocA-S3-*dapB*:pET-28a-ScaA, and QDE-*dapE*-DocA-S3-*dapF*:pET-28a-ScaA based on the intracellular scaffold protein were obtained.

### 3.3 Fermentation analysis of pairwise assemblage of key enzymes for L-lysine synthesis in engineered bacteria

**Figure 6:**
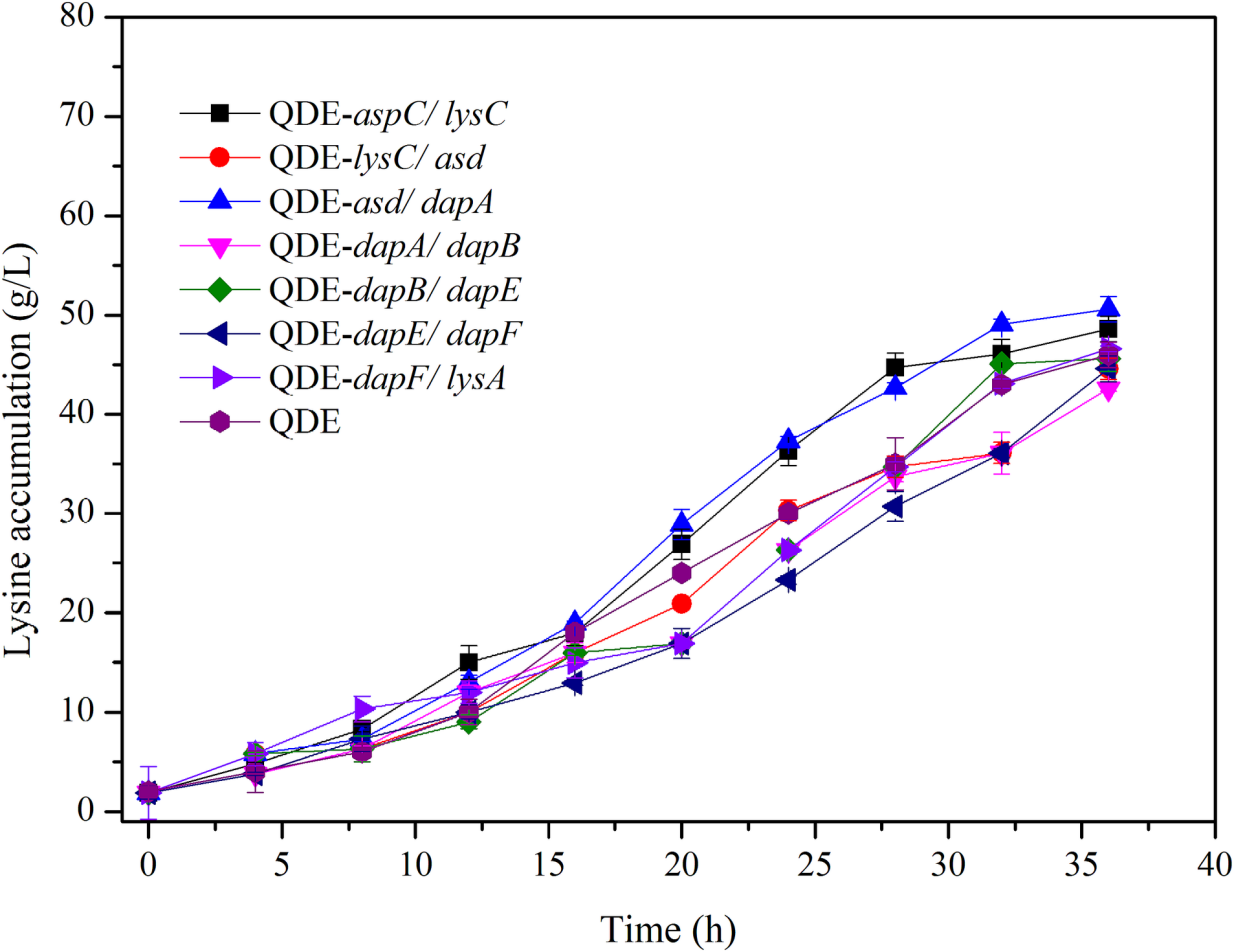
Changes in L-lysine key enzyme genes in pairwise overexpression during fermentation.

Using the L-lysine-producing bacterium *E. coli* QDE as the starting strain, seven control engineered strains with pairing overexpression and unassembled plasmids were obtained. The cumulative L-lysine concentration of *E. coli* QDE from 0 to 36 h was 46.0 g/L, and the conversion rate was 50.9%. The fermentation results showed that the L-lysine concentration of overexpression engineered strain QDE:pET-28a(+)-*aspC/lysC* was 48.6 g/L, 5.6% higher than that of the original strain QDE, and the conversion rate increased from 50.9 to 51.1%. The L-lysine concentration of engineered strain QDE:pET-28a(+)-*asd/dapA* was 50.5 g/L, 9.7% higher than that of the original strain QDE. The L-lysine concentration of the engineered strain QDE:pET-28a(+)-*dapF/lysA* was 46.4 g/L, 0.8% higher than that of the original strain QDE (Figure 6). Although the proximity of related enzymes was not achieved by assembly, the overexpression of the key enzyme genes *aspC* and *lysC*, *asd* and *dapA*, and *dapF* and *lysA* caused a small increase in L-lysine accumulation.

**Figure 7:**
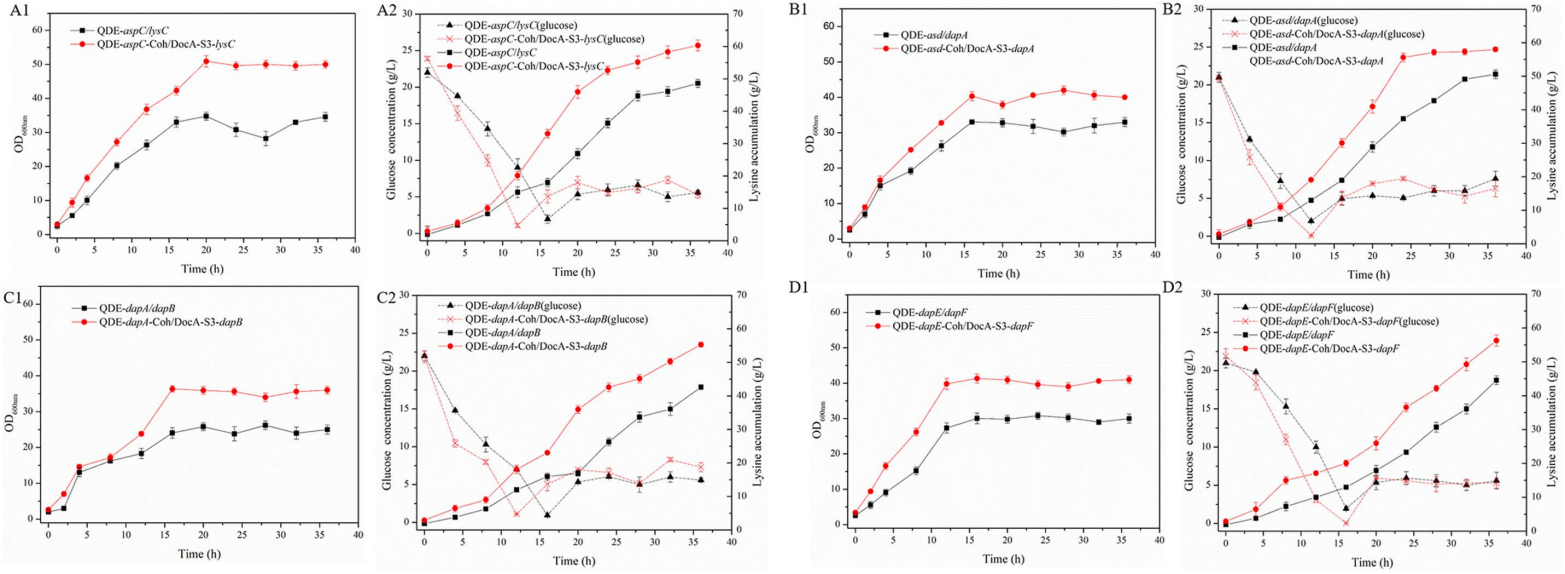
Changes in intracellular pairwise assembly and fermentation process. **(A1–D1)** OD_600_; **(A2–D2)** Glucose and L-lysine concentrations.

The key cellulosome components, DocA-S3 and Coh, were linked to the key enzyme combinations *aspC* and *lysC*, *lysC* and *asd*, *asd* and *dapA*, *dapA* and *dapB*, *dapB* and *dapE*, *dapE* and *dapF*, and *dapF* and *lysA* through linking peptides. Seven strains of key cellulosome skeleton-intracellular pair-to-pair-to-assembly enzymes were identified. During 8–36 h of fermentation, the OD_600_ of the assembled engineered strain QDE:pET-28a(+)-*aspC*-Coh/DocA-S3-*lysC* increased rapidly, which was 2.2–3.4 times that of the control strain QDE:pET-28a(+)-*aspC*/*lysC*. Similarly, the carbon source was rapidly consumed at this stage (Figures 7A1,A2), and L-lysine synthesis efficiency was significantly improved by maintaining the amount of residual sugar. At the end of the fermentation process, the cumulative L-lysine concentration of the engineered strain QDE:pET-28a(+)-*aspC*-Coh/DocA-S3-*lysC* reached 60.3 g/L, 24.1% higher than that of the control strain QDE:pET-28a(+)-*aspC/lysC*. The conversion rate of the overexpression control strains increased from 51.1 to 57.4%. The cumulative L-lysine concentrations of the assembled engineered strains were as follows: QDE:pET-28a(+)-*asd*-Coh/DocA-S3-*dapA*: 58.0 g/L, 14.8% higher than that of the control strain QDE:pET-28a(+)-*asd*/*dapA* (Figures 7B1,B2); QDE:pET-28a(+)-*dapA*-Coh/DocA-S3-*dapB*: 55.3 g/L, 29.8% higher than that of the control strain QDE:pET-28a(+)-*dapA*/*dapB* (Figures 7C1,C2); QDE:pET-28a(+)-*dapE*-Coh/DocA-S3-*dapF*: 56.4 g/L, 26.4% higher than that of the control strain QDE:pET-28a(+)-*dapE*/*dapF* (Figures 7D1,D2).

Therefore, the intracellular pairwise assembly method effectively improves the synergistic effects of most enzymes and increases L-lysine yield. *aspC* and *lysC*, the initial key enzyme genes in the L-lysine synthesis pathway, were assembled using the key component of the cellulosome mutant, DocA-S3, which significantly improved the fermentation efficiency of the strain. The maximum L-lysine concentration in the engineered strain was 60.3 g/L, and the QDE of the engineered strain was 31.1% higher than that of the original strain. The L-lysine conversion rate was 57.4, 12.8% higher than that of the starting strain QDE. A comparison of L-lysine accumulation between the intracellular pair-to-pair-assembled engineered bacteria and the starter strain QDE is shown in Supplementary Figure S4. DocA-S3 significantly improved the fermentation efficiency of the L-lysine-producing strains. Therefore, DocA-S3 could be used to assemble intracellular complexes and improve their synthetic efficiency.

### 3.4 Fermentation analysis of L-lysine synthesis key enzymes based on scaffold protein multi-enzyme assembly in engineered bacteria

Based on the results of pairwise assembly and fermentation of the key enzyme genes in the L-lysine synthesis pathway, four combinations of *aspC* and *lysC*, *asd* and *dapA*, *dapA* and *dapB*, and *dapE* and *dapF* were selected, and the docking protein mutant DocA-S3 was sequentially replaced by a double-fragment homologous substitution in the genome. Intracellular assembly of scaffold protein ScaA was performed. Four strains of cellulosome skeleton-intracellular key enzyme assemblages based on scaffold proteins were obtained, and L-lysine accumulations were compared between unassembled control bacteria and pair-to-pair-assembled engineered bacteria.

**Figure 8:**
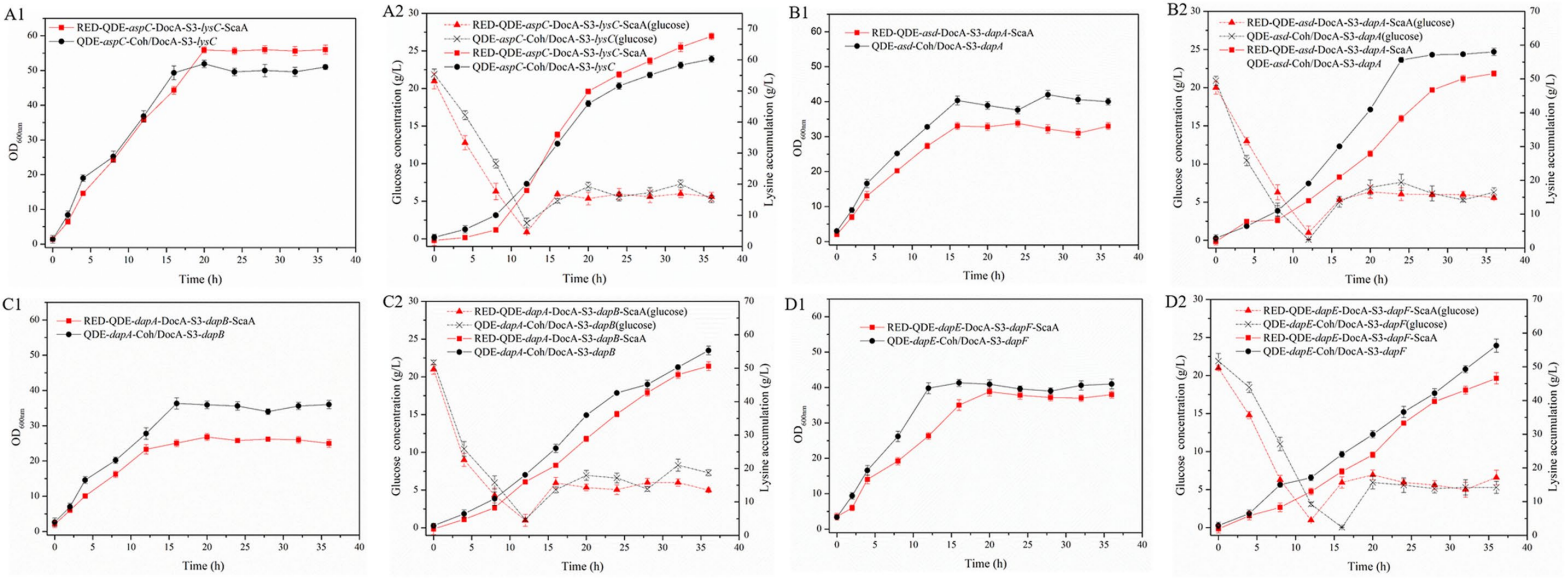
Changes in based on scaffold protein multi-enzyme assembly and fermentation process. **(A1–D1)** OD_600_; **(A2–D2)** Glucose and L-lysine concentrations.

During the 8–36 h of the fermentation process, the L-lysine concentration of multi-enzyme-assembled engineered strain QDE-*aspC*-DocA-S3-*lysC*:pET-28a-ScaA reached 67.6 g/L, 46.9% higher than that of QDE (Figures 8A1,A2); it was 39.1% higher than that of the unassembled control strain QDE:pET-28a(+)-*aspC/lysC* and 12.1% higher than that of the recombinant plasmid pairwise-assembled engineered strain QDE:pET-28a(+)-*aspC*-Coh/DocA-S3-*lysC*. Moreover, the consumption rate of glucose accelerated after 12 h, the conversion rate increased to 59.8%, and the growth and accumulation levels of bacteria were significantly higher than those of the pairwise assembly, increasing by 9.8–12.1%. The cumulative L-lysine concentration of multi-enzyme-assembled engineered strain QDE-*asd*-DocA-S3-*dapA*:pET-28a-ScaA reached 51.3 g/L, 1.6% higher than that of unassembled control strain QDE:pET-28a(+)-*asd*/*dapA*. Compared with that in plasmid pin-to-pair-assembled engineered strain QDE:pET-28a(+)-*asd*-Coh/DocA-S3-*dapA*, the growth rate of bacterial accumulation declined by 11.0%; however, the glucose concentration stabilized after 16 h (Figures 8B1,B2). The cumulative L-lysine concentration of multi-enzyme-assembled engineered strain QDE-*dapA*-DocA-S3-*dapB*:pET-28a-ScaA reached 50.5 g/L, 18.5% higher than that of unassembled control strain QDE:pET-28a(+)-*dapA*/*dapB*. The recombinant plasmid pair-to-pair-assembled engineered strain QDE:pET-28a(+)-*dapA*-Coh/DocA-S3-*dapB* showed aN 8.6% decrease in the cumulative L-lysine concentration (Figures 8C1,C2). The cumulative L-lysine concentration of multi-enzyme-assembled engineered strain QDE-*dapE*-DocA-S3-*dapF*:pET-28a-ScaA reached 46.7 g/L, 4.7% higher than that of unassembled control strain QDE:pET-28a(+)-*dapE/dapF*. Compared with that of plasmid pair-to-pair-assembled engineered strain QDE:pET-28a(+)-*dapE*-Coh/DocA-S3-*dapF*, the reduction was 17.2% (Figures 8D1,D2). These results indicate that the assembly of the two key enzyme genes *aspC* and *lysC* based on the scaffold protein multi-enzyme had the best effect. Similarly, the consumption of the target product L-lysine and the substrate glucose maintained a basic balance, and the product accumulation had more evident advantages than that in the pairwise assembly.

### 3.5 Screening of differential metabolites of assembled engineered bacteria

The overall distribution of metabolite differences between the groups was visualized using the Volcano plot, where each point represented a peak and contained all substances measured in this experiment. Red and blue indicate significantly upregulated and downregulated metabolites, respectively, whereas gray represents a non-significant difference.

**Figure 9:**
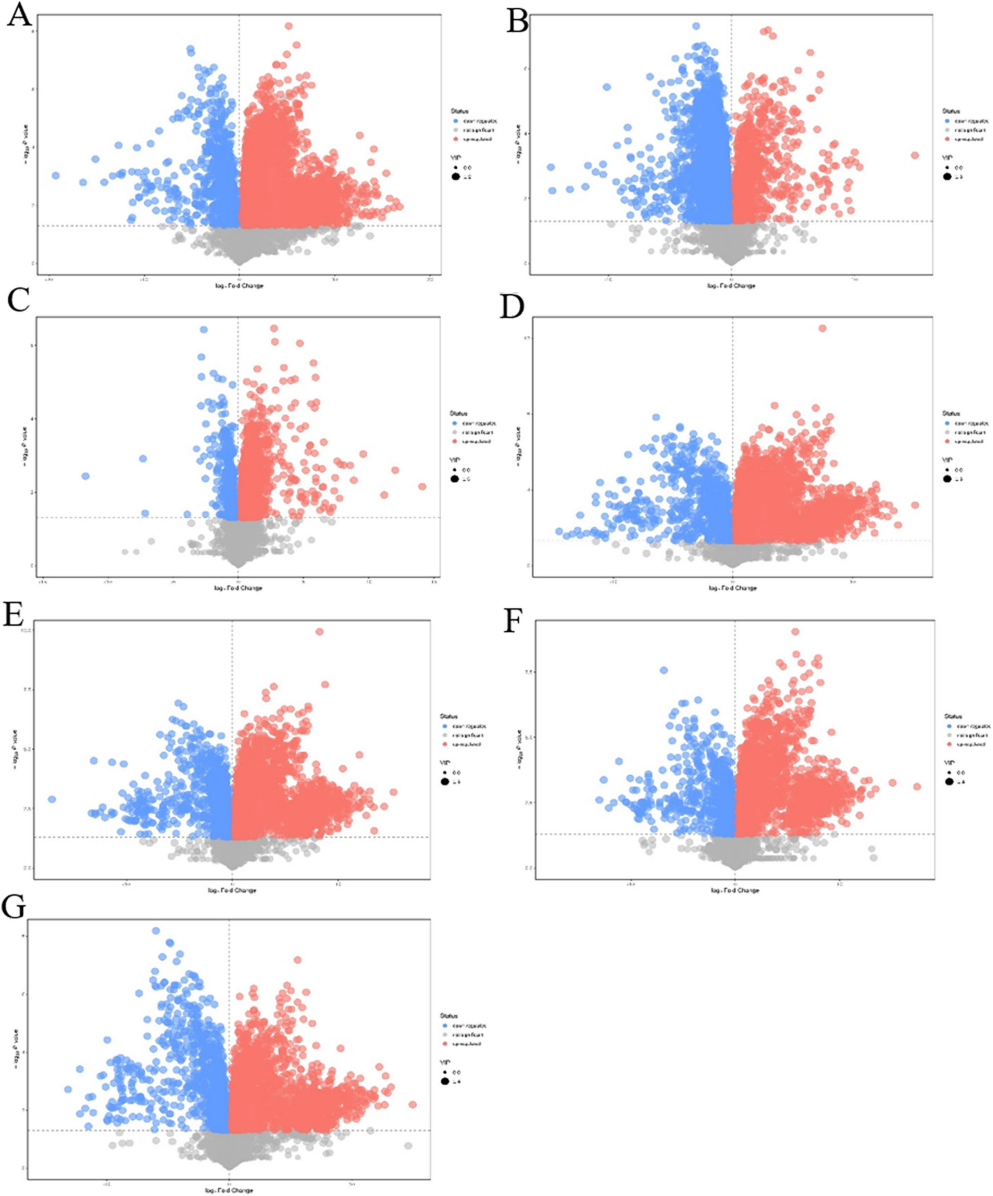
Volcano plot of differential metabolites screening between plasmid pairwise assembly samples. **(A)** Assembled QDE:pET-28a(+)-*aspC*-Coh/DocA-S3-*lysC* and unassembled QDE:pET-28a(+)-*aspC/lysC*; **(B)** Assembled QDE:pET-28a(+)-*lysC*-Coh/DocA-S3-*asd* and unassembled QDE:pET-28a(+)-*lysC/asd*; **(C)** Assembled QDE:pET-28a(+)-*asd*-Coh/DocA-S3-*dapA* and unassembled QDE:pET-28a(+)-*asd/dapA*; **(D)** Assembled QDE:pET-28a(+)-*dapA*-Coh/DocA-S3-*dapB* and unassembled QDE:pET-28a(+)-*dapA/dapB*; **(E)** Assembled QDE:pET-28a(+)-*dapB*-Coh/DocA-S3-*dapE* and unassembled QDE:pET-28a(+)-*dapB/dapE*; **(F)** Assembled QDE:pET-28a(+)-*dapE*-Coh/DocA-S3-*dapF* and unassembled QDE:pET-28a(+)-*dapE/dapF*; **(G)** Assembled QDE:pET-28a(+)-*dapF*-Coh/DocA-S3-*lysA* and unassembled QDE:pET-28a(+)-*dapF/lysA*.

Orthogonal projections to latent structure-discriminant analysis were used, and VIP > 1 and *p*-value<0.05 of the OPLS-DA model were the screening criteria. A total of 5,399 differential metabolites were detected in the intracellular pairwise assembly QDE:pET-28a(+)-*aspC*-Coh/DocA-S3-*lysC* experimental group, among which 4,671 were significantly upregulated and 728 were significantly downregulated (Figure 9A). A total of 4,364 differential metabolites were detected in the intracellular pairwise assembly QDE:pET-28a(+)-*lysC*-Coh/DocA-S3-*asd* experimental group, among which 939 and 3,425 were significantly upregulated and downregulated, respectively (Figure 9B). A total of 2,612 differential metabolites were detected in the intracellular pairwise assembly QDE:pET-28a(+)-*asd*-Coh/DocA-S3-*dapA* experimental group, among which 2,245 and 367 were significantly upregulated and downregulated, respectively (Figure 9C). In the intracellular pairwise assembly QDE:pET-28a(+)-*dapA*-Coh/DocA-S3-*dapB* experimental group, 4,714 differential metabolites were identified, among which 3,900 and 814 were significantly upregulated and downregulated, respectively (Figure 9D). The intracellular pairwise assembly QDE:pET-28a(+)-*dapB*-Coh/DocA-S3-*dapE* experimental group had 4,887 differential metabolites, among which 3,849 and 1,038 were upregulated and downregulated, respectively (Figure 9E). We identified 3,707 differential metabolites in the intracellular pairwise assembly QDE:pET-28a(+)-*dapE*-Coh/DocA-S3-*dapF* experimental group, of which 2,744 and 963 were significantly upregulated and downregulated, respectively (Figure 9F). A total of 3,379 differential metabolites were detected in the intracellular pairwise assembly QDE:pET-28a(+)-*dapF*-Coh/DocA-S3-*lysA* experimental group, with 2,265 and 1,114 showing significant upregulation and downregulation, respectively (Figure 9G).

The combination of *aspC* and *lysC*, *asd* and *dapA*, *dapA* and *dapB*, *dapB* and *dapE*, *dapE* and *dapF*, and *dapF* and *lysA* improved metabolic expression by the pairwise assembly of cellulosome skeleton plasmids. Among them, the pairwise assembly of *aspC* and *lysC*, *asd* and *dapA*, *dapA* and *dapB*, *dapE* and *dapF* caused the most metabolite upregulation. The identified metabolites primarily included amino acids, sugars, and nucleotides.

**Figure 10:**
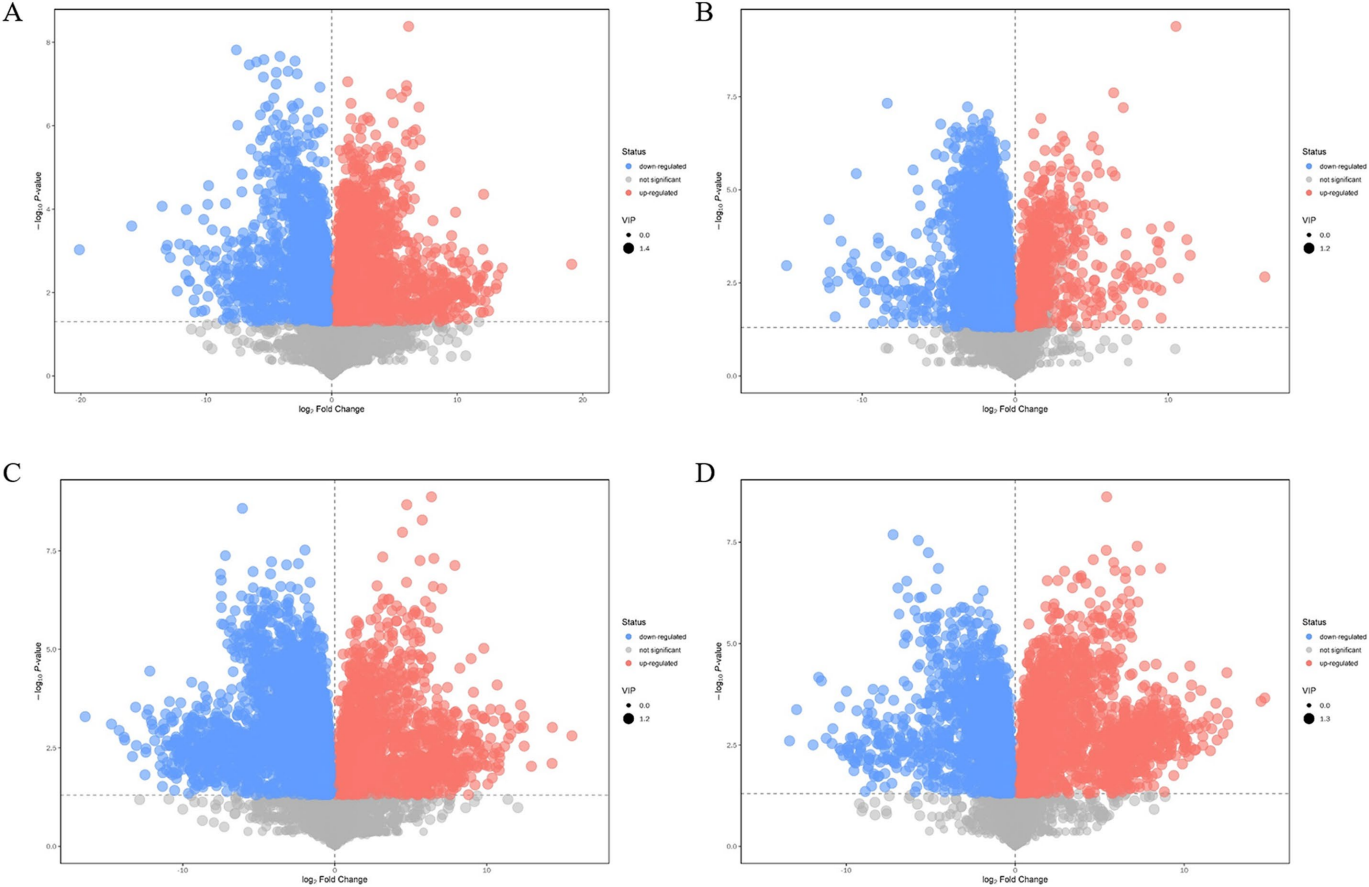
Volcano plot of differential metabolite screening between multi-enzyme assembly samples. **(A)** Multi-enzyme assembly QDE-*aspC*-DocA-S3-*lysC*:pET-28a-ScaA and pairwise assembly QDE:pET-28a(+)-*aspC*-Coh/DocA-S3-*lysC* groups; **(B)** Multi-enzyme assembly QDE-*asd*-DocA-S3-*dapA*:pET-28a-ScaA and pairwise assembly QDE:pET-28a(+)-*asd*-Coh/DocA-S3-*dapA* groups; **(C)** Multi-enzyme assembly QDE-*dapA*-DocA-S3-*dapB*:pET-28a-ScaA and pairwise assembly QDE:pET-28a(+)-*dapA*-Coh/DocA-S3-*dapB* groups; **(D)** Multi-enzyme assembly QDE-*dapE*-DocA-S3-*dapF*:pET-28a-ScaA and pairwise assembly QDE:pET-28a(+)-*dapE*-Coh/DocA-S3-*dapF* groups.

Compared with those of the plasmid pairwise assembly for intracellular L-lysine key enzyme assembly, 3,449 different metabolites were identified in the intracellular scaffold protein-based multi-enzyme assembly QDE-*aspC*-DocA-S3-*lysC*:pET-28a-ScaA group, of which 1777 and 1,672 were significantly upregulated and downregulated, respectively (Figure 10A). A total of 5,000 different metabolites were detected in the intracellular scaffold protein-based multi-enzyme assembly QDE-*asd*-DocA-S3-*dapA*:pET-28a-ScaA group, of which 768 and 4,232 were significantly upregulated and downregulated, respectively (Figure 10B). The intracellular scaffold protein-based multi-enzyme assembly QDE-*dapA*-DocA-S3-*dapB*:pET-28a-ScaA group had 5,054 different metabolites, of which 1,499 and 3,555 were significantly upregulated and downregulated, respectively (Figure 10C). In the intracellular scaffold protein-based multi-enzyme assembly QDE-*dapE*-DocA-S3-*dapF*:pET-28a-ScaA group, 4,536 metabolites were differentially expressed, of which 2059 and 2,477 were significantly upregulated and downregulated, respectively (Figure 10D).

**Figure 11:**
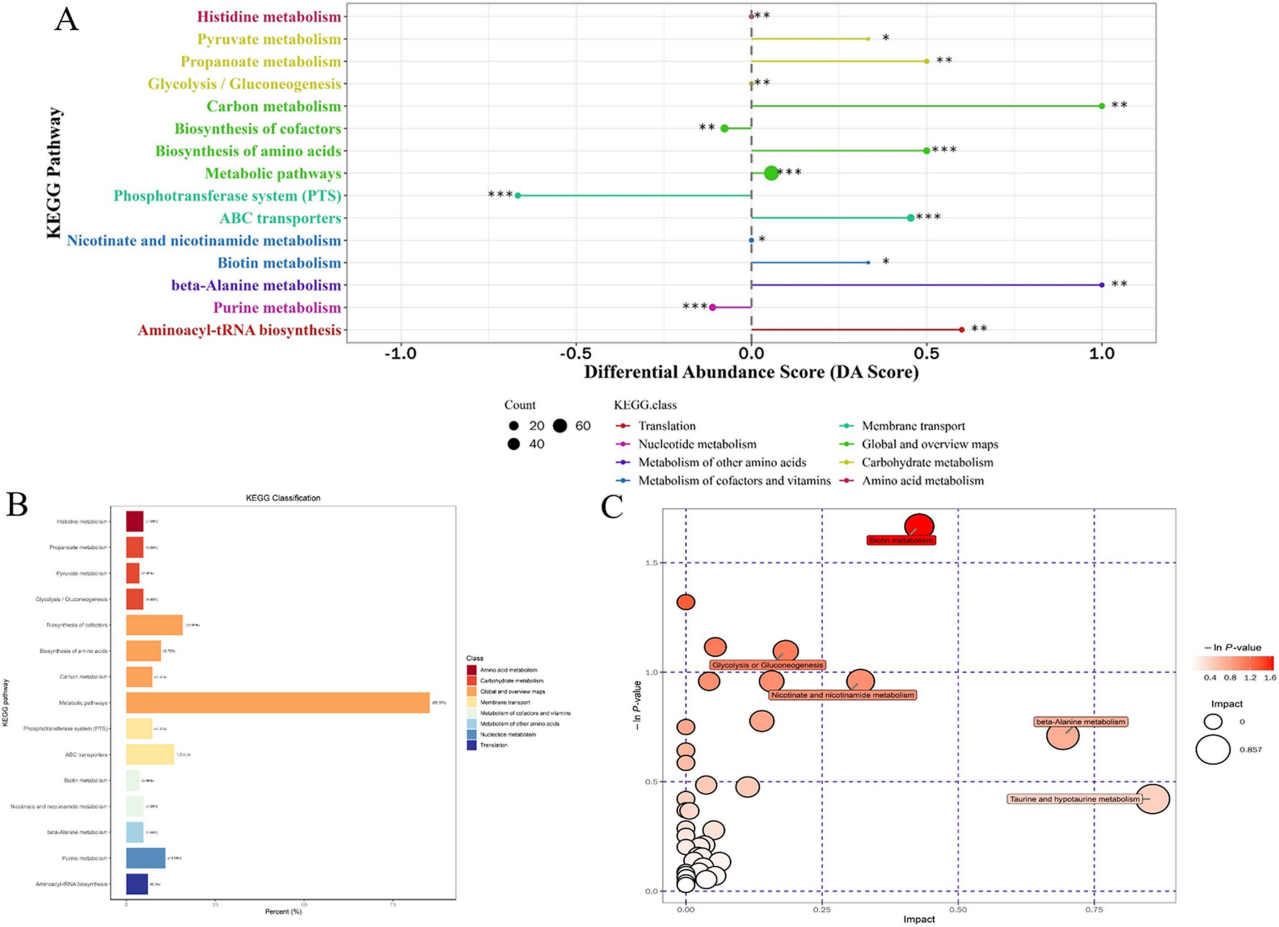
Multienzyme genome assembly of engineered bacteria for *aspC* and *lysC* differential metabolite analysis diagram. **(A)** Metabolic differential abundance scores of multienzyme assembled *aspC* and *lysC* strains; **(B)** KEGG classification of metabolites of metabolic differences between *aspC* and *lysC* strains assembled by multiple enzymes; **(C)** Multienzyme assembly of *aspC* and *lysC* strains of different metabolite pathway analysis.

For the most advantageous intracellular scaffold protein-based multi-enzyme assembly strain QDE-*aspC*-DocA-S3-*lysC*:pET-28a-ScaA, the metabolic pathways covering the most differentiated metabolites were amino acid biosynthesis, ABC transporter, carbon metabolism, *β*-alanine, and aminoacyl-tRNA biosynthesis. Figure 11A shows the differential abundance score. The expression of the differential metabolites annotated in the pathway tended to be up-regulated (Figure 11B). Taurine and hypotaurine metabolism, β-alanine metabolism, and biotin metabolism are major influencing factors in the metabolic pathway, among which biotin metabolism is significantly enriched, as shown in Figure 11C.

### 3.6 Mechanism analysis of the influence of key enzymes on L-lysine synthesis

**Figure 12:**
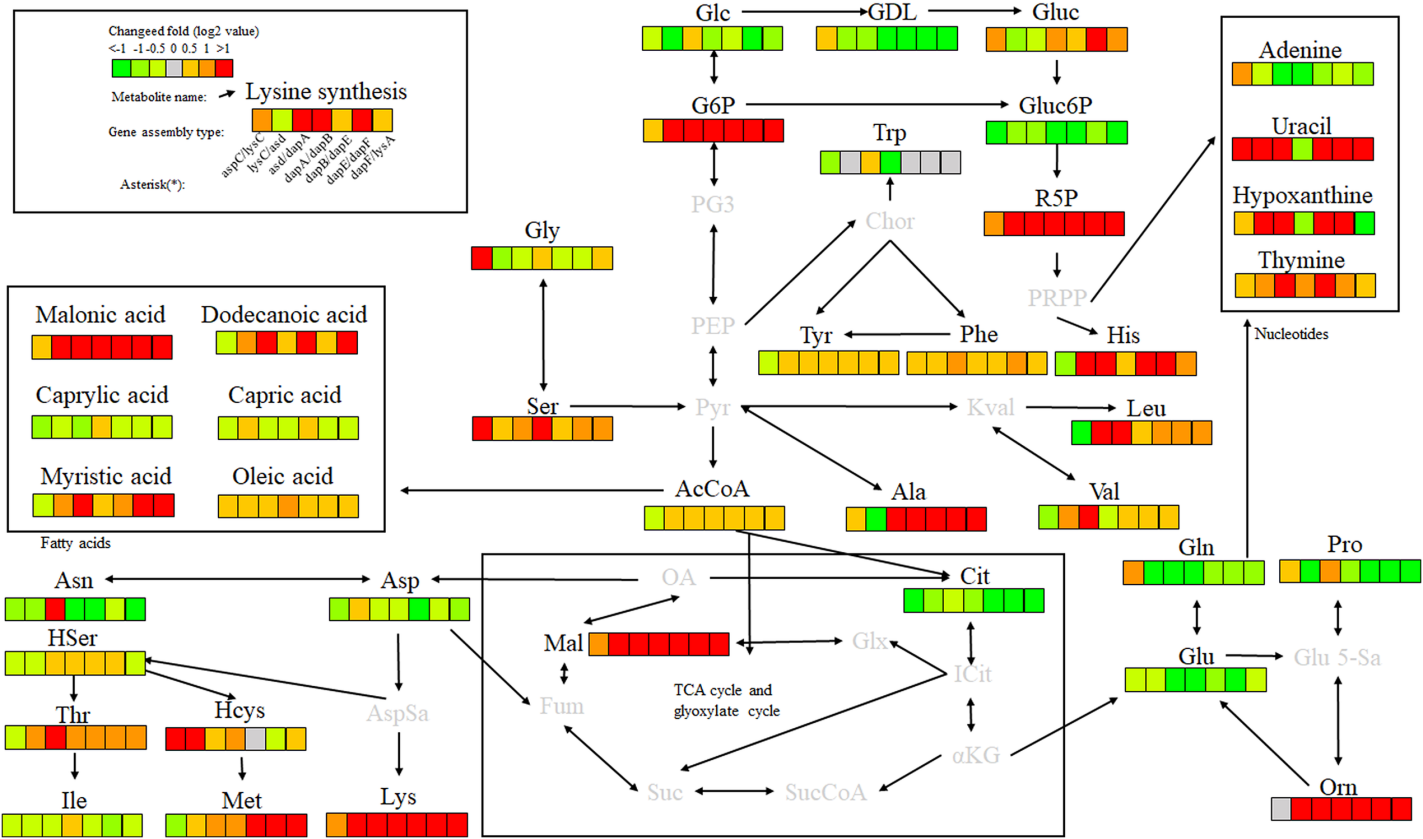
Metabolite analysis of key enzymes for L-lysine synthesis by intracellular pairwise assembly.

Key enzyme genes in the L-lysine fermentation pathway were pair-to-pair assembled with docking protein mutants DocA-S3 and Coh, and 40 core metabolites were identified through metabolomic analysis of major metabolites, which were sequentially located in the metabolic pathways related to L-lysine fermentation in the engineered strain *E. coli* QDE, as shown in Figure 12. The metabolites included amino acids, organic acids, carbohydrates, lipids, lipid molecules, purines, and pyrimidines. After 96 h of fermentation, some of the core metabolites of the engineered strain, based on the L-lysine synthesis key enzyme pairwise assembly of the cellulosome skeleton, were altered compared with those in the original strain *E. coli* QDE. In terms of L-lysine synthesis, the order of efficiency improvement was: QDE:pET-28a(+)-*asd*-Coh/DocA-S3-*dapA* > QDE:pET-28a(+)-*dapA*-Coh/DocA-S3-*dapB* > QDE:pET-28a(+)-*dapE*-Coh/DocA-S3-*dapF* > QDE:pET-28a(+)-*aspC*-Coh/DocA-S3-*lysC* > QDE:pET-28a(+)-*dapB*-Coh/DocA-S3-*dapE* > QDE:pET-28a(+)-*dapF*-Coh/DocA-S3-*lysA* > QDE:pET-28a(+)-*lysC*-Coh/DocA-S3-*asd*. The malic acid contents produced by the eight key enzymes (when combined) *aspC* and *lysC*, *lysC* and *asd*, *asd* and *dapA*, *dapA* and *dapB*, *dapB* and *dapE*, *dapE* and *dapF*, and *dapF* and *lysA* were significantly increased in the TCA cycle stage. The content was 1.5, 3.9, 8.0, 4.7, 2.9, 12.8 and 11.5 times that of the control bacteria *E. coli* QDE, respectively, which promoted the efficient synthesis of multiple amino acids. The Asp-free content of the metabolite L-aspartate was significantly reduced by 25.0–71.4% and was more rapidly applied to the subsequent synthesis of L-lysine as a key precursor, causing a significant increase in L-lysine production. The pair-to-pair assembly of *dapB* and *dapE*, *dapE* and *dapF*, and *dapF* and *lysA* resulted in the accumulation of L-methionine (Met), and the MET was increased by 2.2–2.6 times. The assembly of *asd* and *dapA* successively increases the production of L-aspartate, L-threonine, homoserine, and homocysteine. The substantial Asp consumption in cells resulted in a shortage of precursor supply, requiring more glucose-6-phosphate (G6P) to be supplied for metabolism over time; the G6P content was increased by 1.1, 3.8, 8.4, 4.8, 4.8, 4.8, and 4.8 times, respectively, compared with those in unassembled bacteria, and the subsequent AcCoA and amino acid synthesis precursor substances were supplemented. The metabolic contents of related amino acids, such as L-histidine, L-leucine, L-valine, L-alanine, L-serine, L-glycine, and ornithine, were significantly increased.

In the analysis of related metabolites, the most evident changes before and after assembly were observed in G6P, L-aspartate (Asp), and L-lysine, which are closely related to amino acid metabolism. Intracellular pairwise assembly also affected fatty acid and nucleotide metabolism, with significant increases in malonic, lauric, myristic, and oleic acids. Based on the pair assembly of L-lysine key enzyme strains by cellulosome elements, the operational efficiency of the related metabolic pathways was comprehensively improved, including those of G6P and ribulose 5-phosphate in the glycolysis and pentose phosphate pathways. Similarly, the efficient participation of acetyl-CoA in the metabolic cycle was enhanced. As a significant intermediate metabolite in the metabolism of energy substances, acetyl-CoA integrates sugars, proteins, and fats in the tricarboxylic acid cycle and oxidative phosphorylation, improving the biosynthetic efficiency of subsequent amino acids, sugar alcohols, and their derivatives.

**Figure 13:**
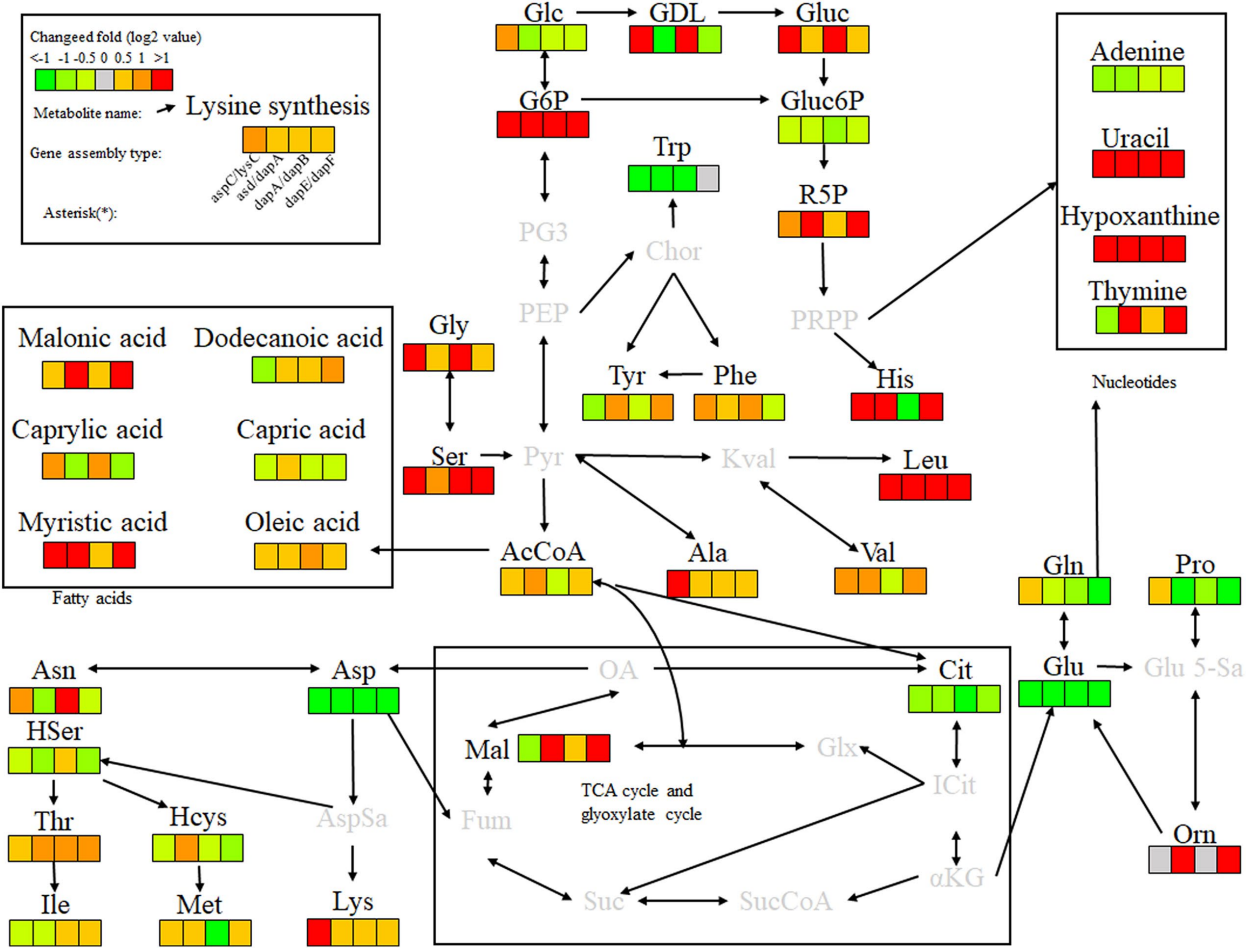
Metabolite analysis of key enzymes for L-lysine synthesis by intracellular multi-enzyme assembly.

Key enzyme combinations of L-lysine synthesis with good pairwise assembly effects, including *aspC* and *lysC*, *asd* and *dapA*, *dapA* and *dapB*, and *dapE* and *dapF* pairwise assemblies, were selected for intracellular scaffold protein-based multi-enzyme assembly, and the effects were compared with those of pairwise assembly. Based on the DocA-S3 intracellular multi-enzyme assembly of key enzymes for L-lysine synthesis, the metabolic efficiency improved in the following order: QDE-*aspC*-DocA-S3-*lysC*:pET-28a-ScaA > QDE-*asd*-DocA-S3-*dapA*:pET-28a-ScaA > QDE-*dapA*-DocA-S3-*dapB*:pET-28a-ScaA > QDE-*dapE*-DocA-S3-*dapF*:pET-28a-ScaA. The scaffold-based multi-enzyme assembly of *aspC* and *lysC*, *asd* and *dapA*, *dapA* and *dapB*, and *dapE* and *dapF* significantly improved the efficiency of glycolysis, the pentose phosphate pathway, and similar amino acid metabolism. For example, the contents of gluconolactone, D-gluconic acid, ribulose 5-phosphate, and G6P were higher in the pentose phosphate pathway, and the G6P contents were increased by 2.2, 4.2, 2.9, and 4.8 times, respectively, compared with those in bacteria with pairwise assembly. The Gluc content of the D-gluconic acid pathway was increased 2.3, 1.4, 6.5, and 1.3 times compared with pairwise-assembled bacteria, and the R5P content of the ribulose 5-phosphate pathway was increased by 2.3, 1.4, 6.5, and 1.3 times compared with pairwise-assembled bacteria, respectively. The L-glycine, L-serine, L-histidine, valine, alanine, and L-leucine contents were also significantly increased. Compared with the effect of pairwise assembly of key enzymes, multi-enzyme assembly based on scaffold proteins had slight effects on the TCA cycle and the subsequent synthesis of related amino acids (Figure 13). For L-lysine, L-asparagine, L-threonine, and methionine, the enhancement was weaker than that of the intracellular pairwise assembly. Because of the expression of scaffold protein repeats, multi-enzyme assemblies based on scaffold proteins have high requirements for the structure and expression level regulation of key enzymes, and the precise enhancement of a certain type of target product requires further investigation.

For the accumulation of the target product, L-lysine, the key enzyme combinations with the most evident effect of scaffold protein-based multi-enzyme assembly were *aspC* and *lysC*, consistent with the conclusion of the intracellular pairwise assembly, indicating that the intracellular self-assembly system based on the key components of cellulosomes promotes the sequential reaction of L-lysine fermentation in *E. coli*. The spatial proximity effect was used to improve the expression efficiency, and the assembly effect of the initial two key enzyme genes, *aspC* and *lysC,* with DocA-S3-Coh, was the most significant.

## 4 Discussion

In this study, an intracellular self-assembly complex was constructed by connecting key enzymes of the L-lysine fermentation pathway, and the “proximity effect” between multi-enzyme complexes was used to improve the transfer efficiency of intermediate metabolites between different catalytic active centers and improve the fermentation intensity. Based on the stable intracellular interaction between the cellulosome docking protein mutant DocA-S3 and the Coh skeleton, the L-lysine fermentation pathway enzymes aspartate aminotransferases *aspC*, *lysC*, *asd*, *dapA*, *dapB*, *dapE*, *dapF,* and *lysA* were studied. The direction of the connection between L-lysine synthetase and the cellulosome element DocA-S3/Coh was determined according to the protein structure, and the location distant from the catalytic activity center of the key enzyme was selected. The intracellular assembly of key cellulosome enzymes for L-lysine synthesis was studied using two different assembly strategies: plasmid pin-to-pair assembly and genome multi-enzyme assembly based on scaffold proteins.

Pairwise assembly of plasmids relies on an efficient intracellular assembly framework for cellulosomes. Using *E. coli* QDE as the starting strain, the DocA-S3 mutant and Coh were linked to *aspC* and *lysC*, respectively, which are key enzyme genes in the L-lysine fermentation pathway, by linking peptides. The binding locations were analyzed according to the structure of each key enzyme and cloned into pET-28a(+) for co-expression. The transcriptional strength of different promoters significantly influences the regulation of the expression of different genes (Farmer and Liao, 2000; Chen and Zeng, 2017; Wu et al., 2018). To identify strong transcriptional regulatory promoters commonly used in *E. coli* (Stöveken et al., 2011; Manfrão-Netto et al., 2019), in-depth studies on Ptrc promoters are needed. Therefore, the trc promoter was used to regulate transcriptional expression and increase the expression of key enzymes involved in pairwise plasmid assembly. Based on the cellulosome skeletons of DocA-S3 and Coh, eight enzymes were pair-assembled and cloned into a plasmid expression vector. This strategy has also been verified in *β*-alanine synthesis, using *Bacillus subtilis* L-aspartate-*α*-decarboxylase (*bspanD*) and *E. coli*-*aspC* to bind to cellulosome elements to improve the catalytic efficiency and expression level of the enzyme (Lu et al., 2023). For L-lysine application, the cumulative L-lysine concentration of the pair-assembled engineered strain QDE:pET-28a(+)-*aspC*-Coh/DocA-S3-*lysC* was 60.3 g/L, which increased by 24.1%. The conversion rate increased from 50.9 to 57.4%. The free content of L-aspartic acid, the key precursor of L-lysine, was reduced 71.4%, which was more rapidly applied to the subsequent synthesis of L-lysine, while the G6P content was increased by up to 8.4 times in the glycolysis process, and the subsequent acetyl-CoA and amino acid synthesis precursors were supplemented over time. In the TCA cycle stage, the malic acid content was up to 12.8 times that of the control bacteria, which promoted the efficient synthesis of various amino acids.

Based on the multi-enzyme assembly of scaffold proteins, optimizing the structure and expression of the scaffold protein ScaA concurrently is essential. In *E. coli* QDE, based on scaffold protein ScaA, intracellular multienzyme assembly was performed for four gene combinations *aspC* and *lysC*, *asd* and *dapA*, *dapA* and *dapB*, and *dapE* and *dapF*. Nine key synthetic enzymes were assembled and expressed simultaneously, and four engineered strains were obtained via gene fragment replacement based on Red homologous recombination. Compared with the pairwise assembly of plasmids, various free enzymes were located close to each other in space to achieve efficient metabolism. The difficulty of multi-enzyme Red homologous recombination is increased at the genomic level when based on scaffold proteins. Because this assembly mode was greatly affected by other gene structures with similar sequences, nine key enzyme genes were assembled concurrently, and the numbers and proportions of the multi-enzyme expression involved were not specifically regulated, resulting in the unstable expression of related enzymes. Only two key enzyme genes, *aspC* and *lysC*, showed better metabolic efficiency than the plasmid pin-pair assembly in the scaffolder-based multi-enzyme assembly. The cumulative L-lysine concentration of multi-enzyme-assembled engineered strain QDE-*aspC*-DocA-S3-*lysC*:pET-28a-ScaA reached 67.6 g/L, 46.9% higher than that of QDE. The TCA cycle and subsequent synthesis of related amino acids, such as L-lysine, L-asparagine, L-threonine, and methionine, were only slightly promoted.

In addition to the two initial key enzyme genes, *aspC* and *lysC*, the improvement in metabolic efficiency of other combinations was weaker than that of the pairwise assembly, indicating that the self-assembly complex constructed on the genome based on scaffold protein multi-enzyme assembly has higher requirements for the structure and expression of key enzymes due to the expression of scaffold protein repeats. Protein engineering modification and fusion protein design can be adopted to optimize the protein structure, while the expression system can be improved through the optimization of promoters and expression vectors. In order to improve the scalability of the system, a modular assembly strategy can be implemented and the engineering transformation of the cell factory can be carried out. In addition, the folding structure of multiple key enzymes in the synthetic pathway is complex, and whether this assembly will affect the interior of enzyme molecules lacks systematic analysis. The random assembly of pathway enzyme genes is not accurate enough, and the site-specific assembly of enzymes on protein scaffolds and their construction strategies still warrant further study.

For the accumulation of L-lysine as the target product, intracellular pairwise assemblies effectively improve the synergistic effect of most enzymes, increasing L-lysine yield. However, for the two key enzyme genes *aspC* and *lysC*, which were the initial key enzyme genes in the L-lysine synthesis pathway in this study, multi-enzyme assembly based on scaffold protein had the best effect, the consumption of the target product L-lysine and the substrate glucose maintained a basic balance, and the product accumulation had more evident advantages than that in the duo assembly. Regardless of the intracellular assembly method, the use of DocA-S3 significantly improved the fermentation efficiency of the production strains. Therefore, DocA-S3 can be used to construct intracellular multi-enzyme self-assembly complexes to improve synthesis efficiency. These results indicate that the intracellular self-assembly system based on the key components of cellulosomes promotes the sequential reaction of L-lysine fermentation, and the spatial proximity effect improves the delivery efficiency of intermediate metabolites, increasing the accumulation of target products. During the amplification and fermentation of actual industrial strains, optimizing the expression of the initial key enzyme genes can promote the synthesis efficiency of the target product and further enhance the effect of subsequent multi-pathway enzyme gene assembly.

## 5 Conclusion

This study focused on the construction of an intracellular self-assembly system of cellulosome DocA-S3/Coh and involved the construction of L-lysine synthetase self-assembled complex in cells via two approaches: pairwise and scaffolding protein-based multi-enzyme assemblies. The L-lysine concentration of scaffold protein-based multi-enzyme-assembled engineered strain QDE-*aspC*-DocA-S3-*lysC*:pET-28a-ScaA reached 67.6 g/L, 46.9% higher than that of QDE. This was 12.1% higher than that of the engineered plasmid pairwise strain QDE:pET-28a(+)-*aspC*-Coh/DocA-S3-*lysC*. The QDE conversion rate increased from 50.9 to 59.8%. The growth and accumulation levels of bacteria were also significantly higher than that of the pair-to-pair assembly, increasing by 9.8–12.1%. The metabolomic analysis confirmed that the free content of L-aspartic acid, a key L-lysine precursor, was reduced, and it was more rapidly applied to subsequent L-lysine synthesis, whereas the content of G6P was increased during glycolysis to timely supplement subsequent AcCoA and amino acid synthesis precursors. The efficient operation of the L-lysine synthesis pathway was improved, more metabolic flow was concentrated, and L-lysine accumulation was increased. The optimal design and efficient assembly of cellulosomes in the cell were realized, and the metabolic pathway enzymes associated with node-substance-limited L-lysine synthesis were assembled at the plasmid and genome level. The assembly and fermentation effects were verified by metabolite statistics, and the feasibility of the multienzyme intracellular self-assembly complex of cellulosome was analyzed, which provided ideas for the construction of other multienzyme assembly and amino acid synthesis cell factories based on cellulosome elements.

## Data Availability

The original contributions presented in the study are included in the article/Supplementary material, further inquiries can be directed to the corresponding authors.
