# Supplementary material for: Intracellular self-assembly and metabolite analysis of key enzymes for L-lysine synthesis based on key components of cellulosomes
Source: Front Microbiol. 2025 Jun 16;16:1596240. doi: 10.3389/fmicb.2025.1596240 (PMC12206723; doi:10.3389/fmicb.2025.1596240)
Supplement: Supplementary file 1 [file Data_Sheet_1.pdf]

Supporting Information for

Intracellular self-assembly and metabolite analysis of key enzymes for L-lysine synthesis based on key components of cellulosomes

Nan Li, Bowen Du, Xiankun Ren, Lu Yang, Peng Du, Piwu Li, Jianbin Wang, Junlin Li, Jing Xiao, Junqing Wang, Ruiming Wang

Junqing Wang, Ruiming Wang
Email: wjqtt.6082@163.com (Junqing Wang), ruiming3k@163.com (Ruiming Wang)

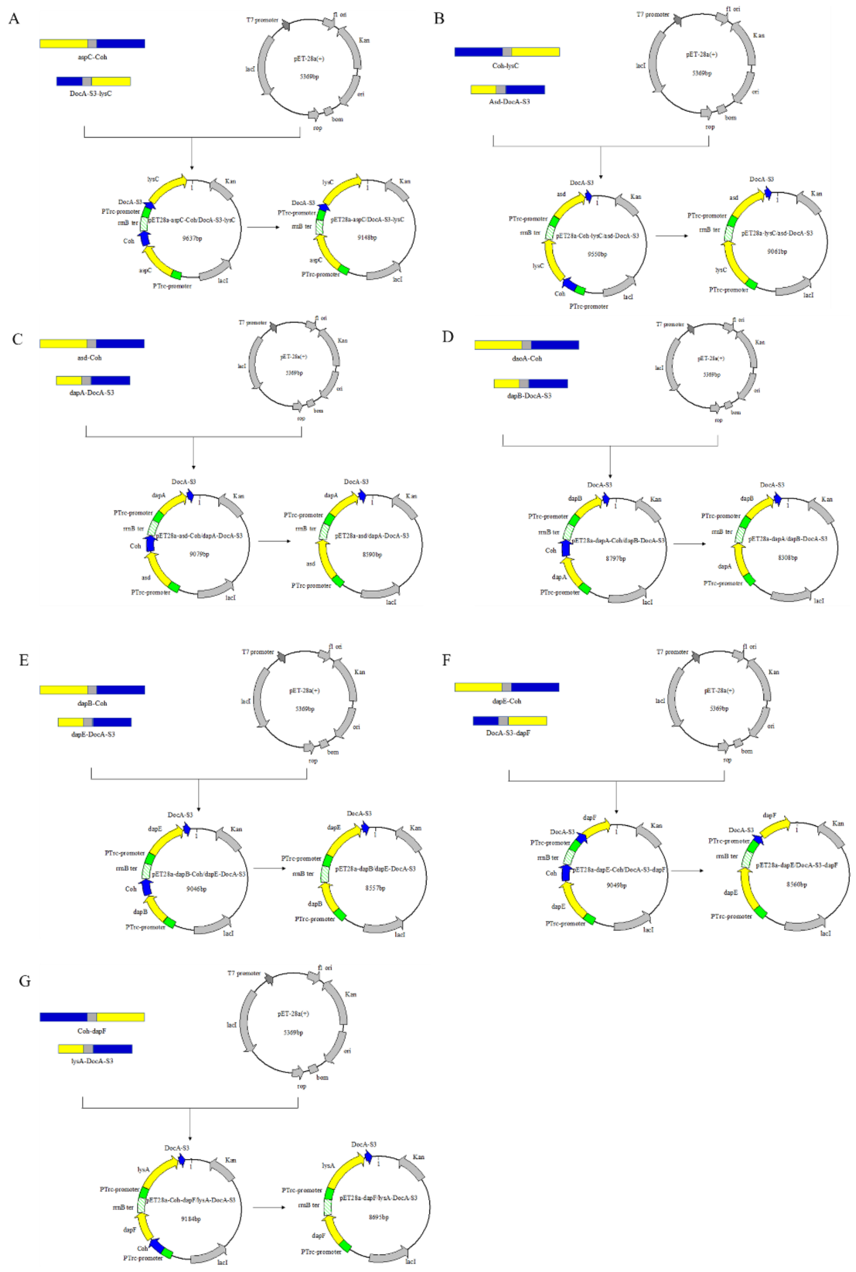

Figure S1. Strategy diagram of pairwise assembled plasmid construction.

(**A**) QDE::pET-28a(+)-*aspC*-Coh/DocA-S3-*lysC* and QDE::pET-28a(+)-*aspC*/*lysC*; (**B**) QDE::pET-28a(+)-*lysC*-Coh/DocA-S3-*asd* and QDE::pET-28a(+)-*lysC*/*asd*; (**C**) QDE::pET-28a(+)-*asd*-Coh/DocA-S3-*dapA* and QDE::pET-28a(+)-*asd*/*dapA*; (**D**) QDE::pET-28a(+)-*dapA*-Coh/DocA-S3-*dapB* and QDE::pET-28a(+)-*dapA*/*dapB;* (**E**) QDE::pET-28a(+)-*dapB*-Coh/DocA-S3-*dapE* and QDE::pET-28a(+)-*dapB*/*dapE*; (**F**) QDE::pET-28a(+)-*dapE*-Coh/DocA-S3-*dapF* and QDE::pET-28a(+)-*dapE*/*dapF*; (**G**) QDE::pET-28a(+)-*dapF*-Coh/DocA-S3-*lysA* and QDE::pET-28a(+)-*dapF*/*lysA*.

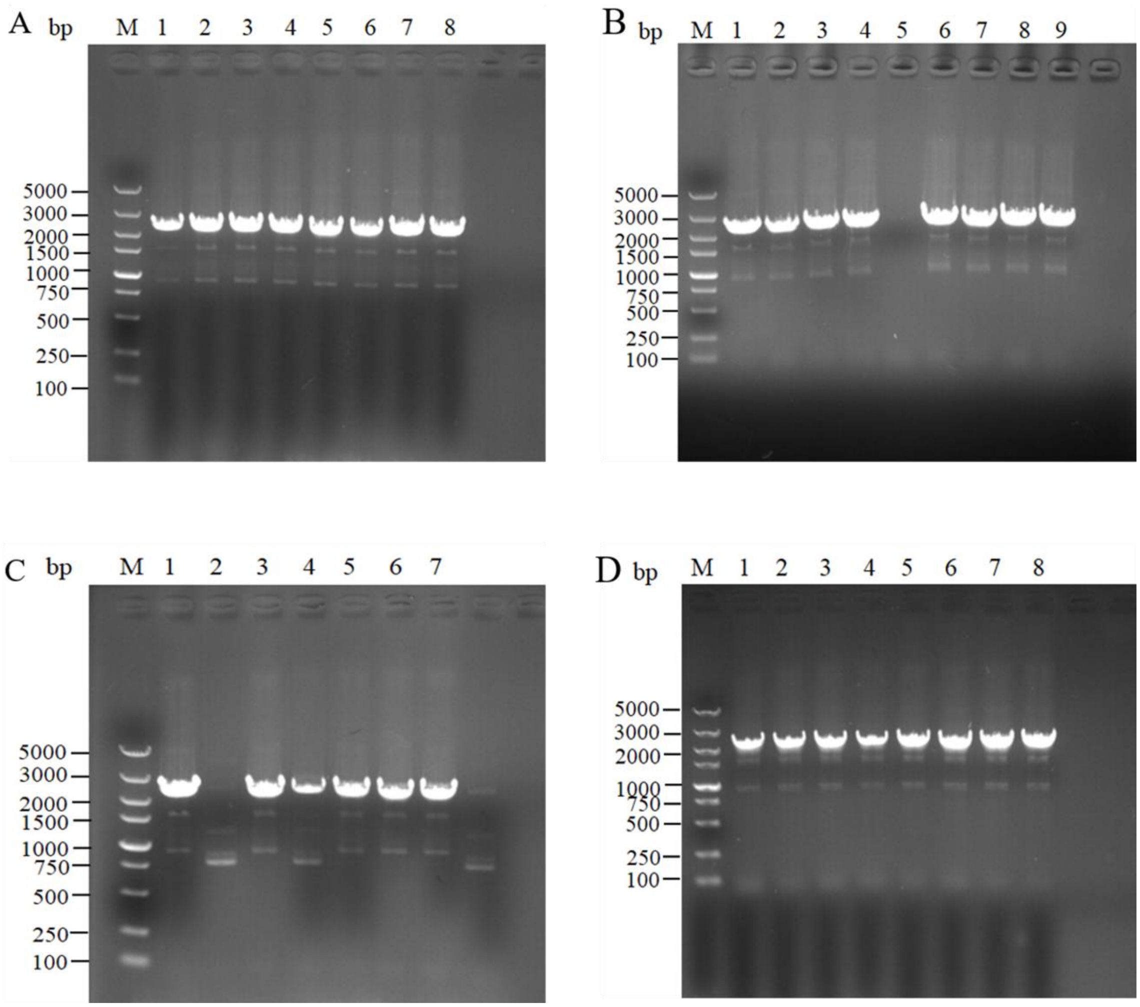

Figure S2. Verification of transformation of fragments of key enzymes assembly frame for L-lysine synthesis.

(**A**) 1-8：2476 bp DNA of assembly frame segment *aspC*1-DocA-S3-FRT-*aspC*Kan-FRT-*aspC*2; (**B**) 1-9：2476 bp DNA of assembly frame segment *asd*1-DocA-S3-FRT-*asd*Kan-FRT-*asd*2; (**C**) 1-7：2476 bp DNA of assembly frame segment *dapA*1-DocA-S3-FRT-*dapA*Kan-FRT-*dapA*2; (**D**) 1-8：2476 bp DNA of assembly frame segment *dapE*1-DocA-S3-FRT-*dapE*Kan-FRT-*dapE*2.

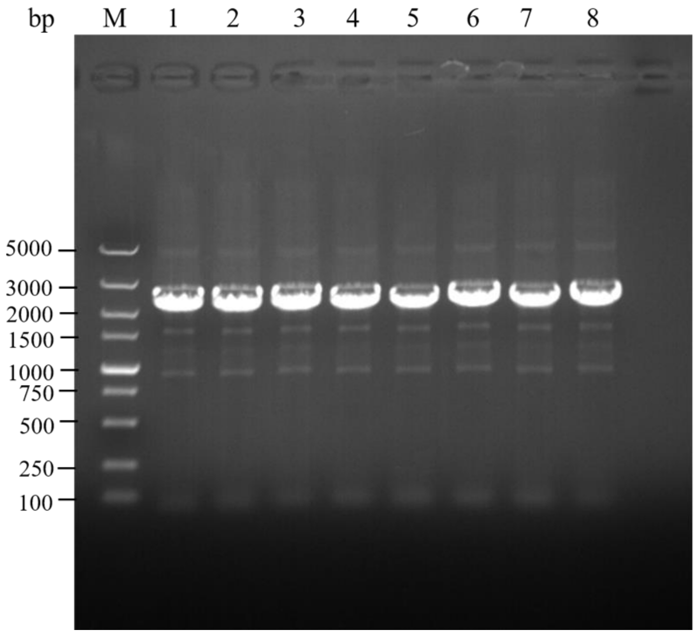

Figure S3. Verification of transformation of fragments of key enzymes assembly frame for L-lysine synthesis. 1-2：2428 bp DNA of assembly frame segment *lysC*1-DocA-S3-FRT-*lysC*Kan-FRT-*lysC*2; 3-4：2428 bp DNA of assembly frame segment *dapA*1-DocA-S3-FRT-*dapA*Kan-FRT-*dapA*2; 5-6：2428 bp DNA of assembly frame segment *dapB*1-DocA-S3-FRT-*dapB*Kan-FRT-*dapB*2; 7-8：2428 bp DNA of assembly frame segment *dapF*1-DocA-S3-FRT-*dapF*Kan-FRT-*dapF*2.

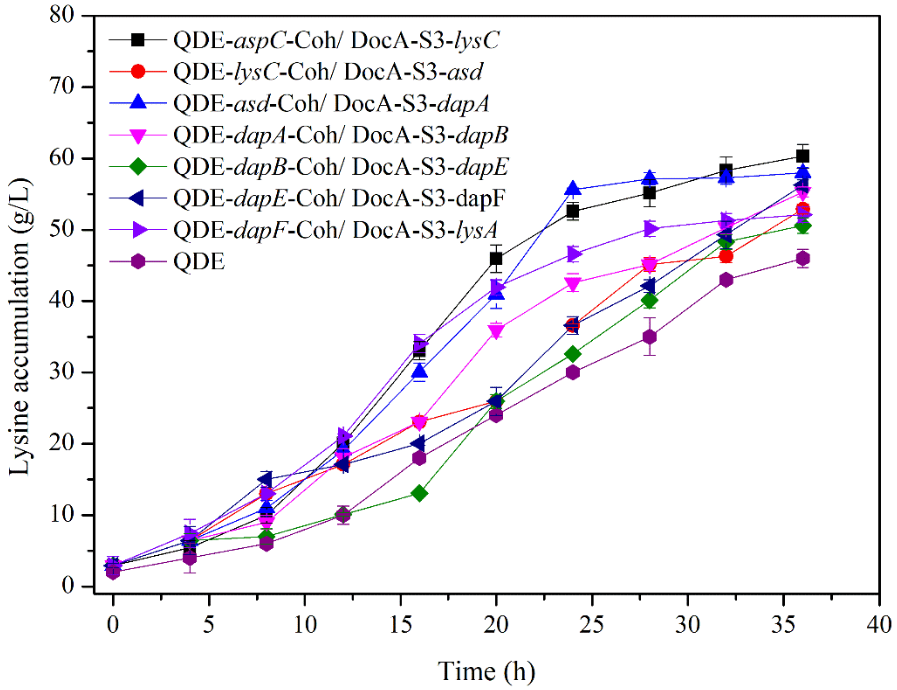

Figure S4. Comparison of L-lysine accumulation between pair-to-assembly engineered bacteria and initial strain QDE.

Table S1 Primers used in the intracellular pairwise assembly

Primer	Sequence（5’→3’）	Size（bp）
aspClC-S3- RF1	AGCGATTGTGGCAGTGCTGTAAGCTGTTTTGGC GGATGAGAGAAGATTTTCAGCCTGATA	60
aspClC-S3- RR1	CTCATCCGCCAAAACAGCTTACAGCACTGCCAC AATCGCTTCGCACAGCGGAGCCATGTT	60
lCasd-S3- RF1	TTTCACACAGGAAACAGACCATGTCTGAAATTG TTGTCTCCAAATTTGGCGGTACCAGCG	60
lCasd-S3- RR1	GAGACAACAATTTCAGACATGGTCTGTTTCCTG TGTGAAATTGTTATCCGCTCACAATTC	60
asddA-S3- RF1	ATGCTTCGTCAACTGGCGTAAGCTGTTTTGGCG GATGAGAGAAGATTTTCAGCCTGATAC	60
asddA-S3- RR1	TCTCATCCGCCAAAACAGCTTACGCCAGTTGAC GAAGCATCCGACGCAGCGGCTCCGCGG	60
dapAdB-S3- RF1	GCTTAAGCATGCCGGTTTGCTGTAAGCTGTTTTG GCGGATGAGAGAAGATTTTCAGCCTG	60
dapAdB-S3- RR1	ATCCGCCAAAACAGCTTACAGCAAACCGGCATG CTTAAGCGCCGCTCTGACCGTCTCACG	60
dapBdE-S3- RF1	GCTTGATCTCAATAGTTTGTAAGCTGTTTTGGCG GATGAGAGAAGATTTTCAGCCTGATA	60
dapBdE-S3- RR1	CTCATCCGCCAAAACAGCTTACAAACTATTGAG ATCAAGCACATCTCGCATATCAAAAAG	60
dapEdF-S3- RF1	TCATGGAACAGCTCGTCGCCTAAGCTGTTTTGG CGGATGAGAGAAGATTTTCAGCCTGAT	60
dapEdF-S3- RR1	TCATCCGCCAAAACAGCTTAGGCGACGAGCTGT TCCATGATACGTTGATACATACGGGCA	60
dapFlA-S3- RF1	TCACACAGGAAACAGACCATGCAGTTCTCGAAA ATGCATGGCCTTGGCAACGATTTTATG	60
dapFlA-S3- RR1	CATGCATTTTCGAGAACTGCATGGTCTGTTTCCT GTGTGAAATTGTTATCCGCTCACAAT	60
T7	TAATACGACTCACTATAGG	19
T7-TER	GCTAGTTATTGCTCAGCGG	19

Table S2 Primers used in Red homologous recombination design

Primer	Sequence (5'→3')	Size (bp)
*aspC*1-F	taagaaggagatataccatggGCTTTCGCGGCTATGCATA	40
*aspC*1-R	tCAGCACTGCCACAATCGCT	20
aspC-S3-F	agcgattgtggcagtgctgAGCGGTGGCGGTAGCGGC	37
aspC-S3-R	ggtccacggagaattcTTAGATGACACGGAGCAGGTAACG	40
aspC-FRT-F	ctaaGAATTCTCCGTGGACCTGCA	24
aspC-FRT -R	tttaatgcGGTACCGAGCTCGGATCCG	27
*aspC*2-F	gagctcggtaccGCATTAAAAACAATGAAGCCCG	34
*aspC*2-R	accagtcatgctagccatatgTTTACAGGTGTTACATTGCCCTGG	45
*lysC*1-F	taagaaggagatataccatggGAATTGTGACTTTGGAAGATTGTAGC	47
*lysC*1-R	cggagaattcAACTACCTCGTGTCAGGGGATCC	33
lysC-S3-F	cgaggtagttGAATTCTCCGTGGACCTGCA	30
lysC-S3-R	atagtaccatGGTACCGAGCTCGGATCCG	29
lysC-FRT-F	gctcggtaccATGGTACTATTAGGGGATGTTGACG	35
lysC-FRT-R	acaatttcagaGCTGCCACCGCTGCCACC	29
*lysC*2-F	ggtggcagcTCTGAAATTGTTGTCTCCAAATTTG	34
*lysC*2-R	accagtcatgctagccatatgCAGCGCGGCCAGTTCCGC	39
*asd*1-F	taagaaggagatataccatggGAACGCAAAGTCACAACCTTAACC	45
*asd*1-R	ctCGCCAGTTGACGAAGCATCC	22
asd-S3-F	atgcttcgtcaactggcgAGCGGTGGCGGTAGCGGC	36
asd-S3-R	ggtccacggagaattcTTAGATGACACGGAGCAGGTAACG	40
asd-FRT-F	ctaaGAATTCTCCGTGGACCTGCA	24
asd-FRT-R	ataaagaGGTACCGAGCTCGGATCCG	26
*asd*2-F	cgagctcggtaccTCTTTATTCATTAAATCTGGGGCG	37
*asd*2-R	accagtcatgctagccatatgTATGTCTCCTCACCGTCTGGTCG	44
*dapA*1-F	taagaaggagatataccatggACTGACCTGCCGCAAATTCTG	42
*dapA*1-R	tCAGCAAACCGGCATGCTT	19

dapA-S3-F	taagcatgccggtttgctgAGCGGTGGCGGTAGCGGC	37
dapA-S3-R	ggtccacggagaattcTTAGATGACACGGAGCAGGTAACG	40
dapA-FRT-F	ctaaGAATTCTCCGTGGACCTGCA	24
dapA-FRT-R	tctccctaaactGGTACCGAGCTCGGATCCG	31
*dapA*2-F	tcggtaccAGTTTAGGGAGATTTGATGGCTTAC	33
*dapA*2-R	accagtcatgctagccatatgTGATAACGACCACGATACTGCTCG	45
*dapB*1-F	taagaaggagatataccatggGACGAAGCCGGTAAACAAGC	41
*dapB*1-R	ccgccaccgctCAAATTATTGAGATCAAGTACATCTCGC	39
dapB-S3-F	aataatttgAGCGGTGGCGGTAGCGGC	27
dapB-S3-R	ggtccacggagaattcTTAGATGACACGGAGCAGGTAACG	40
dapB-FRT-F	ctaaGAATTCTCCGTGGACCTGCA	24
dapB-FRT-R	tattttgtggGGTACCGAGCTCGGATCCG	29
*dapB*2-F	gctcggtaccCCACAAAATATTTGTTATGGTGCAA	35
*dapB*2-R	accagtcatgctagccatatgGAAACTGGGTTCCGTCTTCCA	42
*dapE*1-F	taagaaggagatataccatggCCTTTCCTTAATGAATTAGTGGCTATT	48
*dapE*1-R	ctGGCGACGAGCTGTTCCATG	21
dapE-S3-F	atggaacagctcgtcgccAGCGGTGGCGGTAGCGGC	36
dapE-S3-R	ggtccacggagaattcTTAGATGACACGGAGCAGGTAACG	40
dapE-FRT-F	ctaaGAATTCTCCGTGGACCTGCA	24
dapE-FRT-R	ttgcagaaccactcaGGTACCGAGCTCGGATCCG	34
*dapE*2-F	gtaccTGAGTGGTTCTGCAAGAGGAAAT	28
*dapE*2-R	accagtcatgctagccatatgGTGACGTGACGCTGGATATCG	42
*dapF*1-F	taagaaggagatataccatggGTTTACCGAGCGAGCATCCTC	42
*dapF*1-R	ggtccacggagaattcCATTTACTCCAATCACGCGGG	37
dapF-S3-F	aatgGAATTCTCCGTGGACCTGCA	24
dapF-S3-R	atagtaccatGGTACCGAGCTCGGATCCG	29
dapF-FRT-F	gctcggtaccATGGTACTATTAGGGGATGTTGACG	35

dapF-FRT-R	ttttcgagaactgGCTGCCACCGCTGCCACC	31
*dapF*2-F	tggcagcCAGTTCTCGAAAATGCATGGC	28
*dapF*2-R	accagtcatgctagccatatgATCGACATCATCGACCTGAATCA	44
*lysA*1-F	taagaaggagatataccatggCAAATCGCCCGCCATTTG	39
*lysA*1-R	tAAGCAATTCCAGCGCCAGT	20
lysA-S3-F	actggcgctggaattgcttAGCGGTGGCGGTAGCGGC	37
lysA-S3-R	ggtccacggagaattcTTAGATGACACGGAGCAGGTAACG	40
lysA-FRT-F	ctaaGAATTCTCCGTGGACCTGCA	24
lysA-FRT-R	actaaccgcagGGTACCGAGCTCGGATCCG	30
*lysA*2-F	ctcggtaccCTGCGGTTAGTCGCTGGTTG	29
*lysA*2-R	accagtcatgctagccatatgTATCTGTGCTCTAACCACTCTATTTCTG	49
frame *aspC*-F	gctttcgcggctatgcataaag	22
frame *aspC*-R	tttacaggtgttacattgccctgg	24
frame *lysC*-F	gaattgtgactttggaagattgtagcgc	28
frame *lysC*-R	cagcgcggccagttcc	16
frame *asd*-F	gaacgcaaagtcacaaccttaaccc	25
frame *asd*-R	tatgtctcctcaccgtctggt	21
frame *dapA*-F	actgacctgccgcaaattctg	21
frame *dapA*-R	tgataacgaccacgatactgctcg	24
frame *dapB*-F	gacgaagccggtaaacaagca	21
frame *dapB*-R	gaaactgggttccgtcttccaga	23
frame *dapE*-F	cctttccttaatgaattagtggctattgagtgg	33
frame *dapE*-R	gtgacgtgacgctggatatcg	21
frame *dapF*-F	gtttaccgagcgagcatcct	20
frame *dapF*-R	atcgacatcatcgacctgaatcacg	25
frame *lysA*-F	caaatcgcccgccatttgg	19
frame *lysA*-R	tatctgtgctctaaccactctatttctgac	30
YpKD46-F	atgagtactgcactcgcaacg	21
YpKD46-R	tcatgctgccaccttctgc	19
YpCP20-F	ttacgccccgccctgc	16

YpCP20-R	atggagaaaaaaatcactggatataccacc	30

Table S3 Validation primers used in this chapter

Primer	Sequence (5'→3')	Size (bp)
aspC-F	aaatatgcggccgcGCTTTCGCGGCTATGCATAA	34
frt-R	tttatagcggccgcGGTACCGAGCTCGGATCCGCTA	36
lysC-F	aaatatgcggccgcCGCGCAGGTGGTACGCTG	32
asd-F	aaatatgcggccgcGAACGCAAAGTCACAACCTTAAC	37
dapA-F	aaatatgcggccgcACTGACCTGCCGCAAATTCTG	35
dapB-F	aaatatgcggccgcGACGAAGCCGGTAAACAAGCAA	36
dapE-F	aaatatgcggccgcCCTTTCCTTAATGAATTAGTGGCTA	39
dapF-F	aaatatgcggccgcGTCACCGATGATGATCTGGTCC	36
lysA-F	aaatatgcggccgcCAAATCGCCCGCCATTTGGG	34
YZ-*aspC*-F	TACTCTAAAAACTTTGGCCTGTACAACG	28
YZ-*aspC* -R	CAGCACTGCCACAATCGCTTC	21
YZ-DocA-S3-R	GACACGGAGCAGGTAACGAGACA	23
YZ-*lysC*-F	GCCGCTGTTCCGCGCTCTGGC	21
YZ-*lysC* -R	CTCAAACAAATTACTATGCA	20
YZ-*asd*-F	TGCCGGTGGATAACTTTGGCGT	20
YZ-*asd*-R	AGTTGACGAAGCATCCGACGC	22
YZ-*dapA*-F	TACTGGCTGCGATCTGCTCCC	21
YZ-*dapA*-R	CGGCATGCTTAAGCGCCGCTC	21
YZ-*dapB*-F	ATATTGCGATTGTCTTTGCT	21
YZ-*dapB*-R	ATTATTGAGATCAAGTACATC	21
YZ-*dapE*-F	GGGCAATGAATTCTTCCCGGCG	21
YZ-*dapE*-R	CGACGAGCTGTTCCATGATAC	22
YZ-*dapF*-F	ACCCAACTTCGAACCTTCCGC	21
YZ-*dapF*-R	AGATGAATAAATCCGTCGTAG	21
YZ-*lysA*-F	ATTGAACCGGGTCGCTTCCTGG	21
YZ-*lysA*-R	AATTCCAGCGCCAGTAATTCTTCGA	22
YZ-DocA-S3-F	GTACTATTAGGGGATGTTGAC	25

YZ-frt-F	ATTCTCCGTGGACCTGCAGTT	21
YZ-frt-R	CCGAGCTCGGATCCGCTAGC	20
